# Electrospun wound dressings containing bioactive natural products: physico-chemical characterization and biological assessment

**DOI:** 10.1186/s40824-021-00223-9

**Published:** 2021-07-16

**Authors:** Athanasios S. Arampatzis, Konstantinos N. Kontogiannopoulos, Konstantinos Theodoridis, Eleni Aggelidou, Angélique Rat, Anne Willems, Ioannis Tsivintzelis, Vassilios P. Papageorgiou, Aristeidis Kritis, Andreana N. Assimopoulou

**Affiliations:** 1 Laboratory of Organic Chemistry, School of Chemical Engineering, Aristotle University of Thessaloniki (AUTh), 54124 Thessaloniki, Greece; 2 Natural Products Research Center of Excellence (NatPro-AUTh), Center of Interdisciplinary Research and Innovation of Aristotle University of Thessaloniki (CIRI-AUTh), 57001 Thessaloniki, Greece; 3 Department of Physiology and Pharmacology, Faculty of Health Sciences, School of Medicine, Aristotle University of Thessaloniki (AUTh), 54124 Thessaloniki, Greece; 4 cGMP Regenerative Medicine Facility, Department of Physiology and Pharmacology, Faculty of Health Sciences, School of Medicine, Aristotle University of Thessaloniki (AUTh), 54124 Thessaloniki, Greece; 5 Laboratory of Microbiology, Department of Biochemistry and Microbiology, Faculty of Sciences, Ghent University, 9000 Ghent, Belgium; 6 Physical Chemistry Laboratory, School of Chemical Engineering, Aristotle University of Thessaloniki (AUTh), 54124 Thessaloniki, Greece

**Keywords:** Electrospinning, Drug delivery, Nanofibers, Alkannin, Shikonin, Skin tissue engineering, Wound dressings, Wound healing

## Abstract

**Background:**

Current research on skin tissue engineering has been focusing on novel therapies for the effective management of chronic wounds. A critical aspect is to develop matrices that promote growth and uniform distribution of cells across the wound area, and at the same time offer protection, as well as deliver drugs that help wound healing and tissue regeneration. In this context, we aimed at developing electrospun scaffolds that could serve as carriers for the bioactive natural products alkannin and shikonin (A/S).

**Methods:**

A series of polymeric nanofibers composed of cellulose acetate (CA) or poly(ε-caprolactone) (PCL) and varying ratios of a mixture of A/S derivatives, has been successfully fabricated and their physico-chemical and biological properties have been explored.

**Results:**

Scanning electron microscopy revealed a uniform and bead-free morphology for CA scaffolds, while for PCL beads along the fibers were observed. The average diameters for all nanofibers ranged between 361 ± 47 and 487 ± 88 nm. During the assessment of physicochemical characteristics, CA fiber mats exhibited a more favored profile, while the assessment of the biological properties of the scaffolds showed that CA samples containing A/S mixture up to 1 wt.% achieved to facilitate attachment, survival and migration of Hs27 fibroblasts. With respect to the antimicrobial properties of the scaffolds, higher drug-loaded (1 and 5 wt.%) samples succeeded in inhibiting the growth of *Staphylococcus epidermidis and S. aureus* around the edges of the fiber mats. Finally, carrying out a structure-activity relationship study regarding the biological activities (fibroblast toxicity/proliferation and antibacterial activity) of pure A/S compounds – present in the A/S mixture – we concluded that A/S ester derivatives and the dimeric A/S augmented cell proliferation after 3 days, whereas shikonin proved to be toxic at 500 nM and 1 μM and alkannin only at 1 μM. Additionally, alkannin, shikonin and acetyl-shikonin showed more pronounced antibacterial properties than the other esters, the dimeric derivative and the A/S mixture itself.

**Conclusions:**

Taken together, these findings indicate that embedding A/S derivatives into CA nanofibers might be an advantageous drug delivery system that could also serve as a potential candidate for biomedical applications in the field of skin tissue engineering.

**Supplementary Information:**

The online version contains supplementary material available at 10.1186/s40824-021-00223-9.

## Background

Skin is the human body’s largest organ serving as a protective barrier between the interior and exterior environment. Wounds may occur when there is a loss of the skin integrity resulting in minor or major disabilities based on the severity of the trauma. Wound healing primarily aims at closing the wound as fast as possible and creating a functional and aesthetically satisfactory scar [1].

An appropriate treatment strategy for successful wound healing is the implementation of wound dressings. Their importance and necessity can be illustrated by the numbers in the global wound care market that is estimated to reach $26.24 billion by the end of 2023 [2]. Electrospun fibers have gained widespread importance in the tissue engineering and drug delivery field due to their remarkable characteristics that make them an appealing dressing material for both acute and – especially – chronic wounds (e.g. diabetic foot ulcers, pressure ulcers and venous leg ulcers). The building blocks of electrospun fibers are biocompatible polymers and can be divided into two main categories: natural and synthetic polymers. Electrospun dressings are capable of incorporating and releasing a variety of therapeutic agents that are tailored based on the type and composition of the materials in the fibers, both polymer and active ingredient [3].

Cellulose acetate (CA) is a highly biodegradable, non-toxic and biocompatible material having a wide array of applications in biomedical fields. Particularly, electrospun CA nanofibers have been used in skin and wound healing applications, as well as carriers for delivering drugs topically/transdermally [4]. Poly(ε-caprolactone) (PCL) is a well-known Food and Drug Administration (FDA)-approved, synthetic polymer with good biocompatibility and mechanical properties that has been widely used for biomedical applications [5]. Added to that, when PCL is used as a vehicle for entrapping active compounds it can prolong their release, thus increasing their therapeutic index [6].

Alkannin and Shikonin (A/S) are bioactive natural products biosynthesized in the roots of several boraginaceous plants (e.g. *Alkanna tinctoria* Tausch, *Lithospermum erythrorhizon* Siebold & Zucc., *Arnebia euchroma* I. M. Johnst.). Apart from a number of important pharmacological qualities, such as antitumor, antimicrobial, antioxidant and anti-inflammatory activities, these molecules have been primarily well-known for their strong wound healing and regenerative properties [7, 8]. Prof. Papageorgiou was the first one (1978) to establish the wound healing and regenerating activities of these active pharmaceutical ingredients (APIs) through multiple clinical studies delivering beneficial effects to severe chronic wounds [9]. As a result, several international patents have been granted to Prof. Papageorgiou.

Since the last decade, there have been a few studies of different research groups that attempted to embed shikonin, alkannin or related plant extracts (e.g. *L. erythrorhizon* extract) into electrospun nanofibers and investigate their physicochemical and biological properties. Han et al. [10] first fabricated electrospun PCL/poly(trimethylene carbonate) (PTMC) nanofibers loaded with shikonin. The results indicated that the antioxidant activity of shikonin was retained before and after electrospinning, while the shikonin-loaded fiber mats hindered the growth of two bacterial species, *Staphylococcus aureus* and *Escherichia coli*. Our group – at the Organic Chemistry Laboratory of the School of Chemical Engineering at AUTh – for first time used different polymers, such as poly(lactic-L-acid) (PLLA), poly(lactic-co-glycolic acid) (PLGA) and CA as carriers for shikonin or a mixture of A/S derivatives isolated from *A. tinctoria* roots, showing that high drug entrapment efficiencies and appropriate release profiles were obtained for all polymer types [11]. Another group produced polyvinyl alcohol (PVA) nanofibers with *L. erythrorhizon* extract, by means of electrospinning. The indirect cell viability assay of the drug-loaded nanofibrous scaffolds showed that the viability of L929 fibroblasts was significantly enhanced [12]. Two more recent studies shed light on the wound healing effect of electrospun lithospermi radix extract-containing scaffolds [13, 14]. The authors constructed a bilayer scaffold that proved to be non-toxic for L929 fibroblasts, demonstrated a good in vitro cell attachment and achieved the highest wound recovery rate when compared with other dressings in vivo. More recently, our group published a study reporting the use for the first time of poly[(R)-3-hydroxybutyric acid] (PHB) as a biomaterial for fabricating electrospun scaffolds containing a mixture of A/S derivatives. The API-loaded e-spun samples showed satisfactory physicochemical characteristics, while the in vitro biological assessment of the A/S mixture-containing fiber mats revealed the induction of Hs27 fibroblasts’ attachment and proliferation, as well as the antibacterial activity against *S. aureus* and *S. epidermidis* [15]*.*

This study is a continuation of our previous works in an effort to develop a potential bioactive wound dressing with sustained drug release for the effective management of wounds; it aims at evaluating the in vitro biocompatibility of electrospun CA and PCL nanofibers loaded with a mixture of A/S derivatives (A/S mixture) as API. In the present work, CA and PCL nanofiber meshes loaded with A/S mixture in different concentrations were fabricated via electrospinning for potential wound healing applications. The prepared scaffolds were characterized by scanning electron microscopy (SEM) and differential scanning calorimetry (DSC). Additionally, drug entrapment and release kinetics of the A/S mixture-loaded scaffolds were examined. Nanofibers were also assessed for their cytocompatibility using dermal fibroblasts Ηs27 in terms of cell attachment and morphology, cell proliferation and migration, as well as for their antimicrobial effect against *Staphylococcus aureus* and *S. epidermidis*. Finally, a structure-activity relationship (SAR) study was undertaken regarding the effect of individual A/S derivatives contained in the A/S mixture on both the viability of Hs27 cells, as well as the antibacterial activity against staphylococci*.*

## Materials and methods

### Chemicals and reagents

**Fig. 1:**
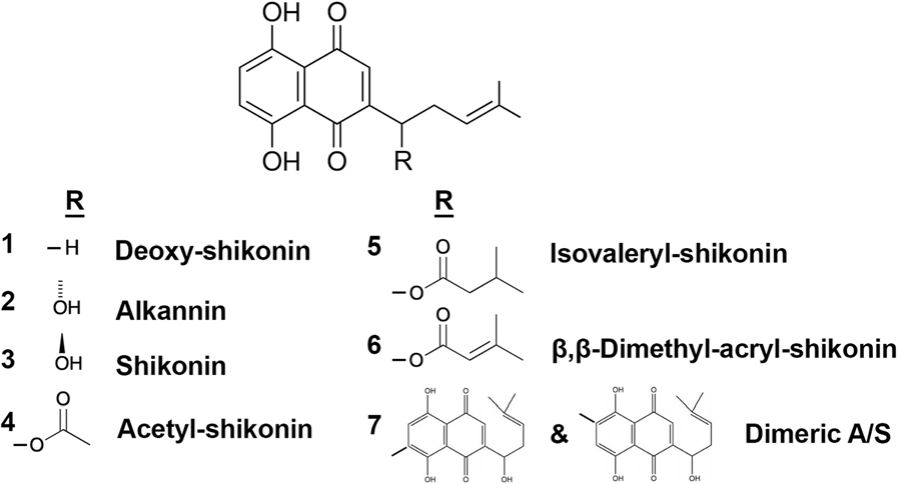
Chemical structures of A/S derivatives

The A/S mixture was isolated from *A. tinctoria* roots obtained from Soft-N-Supple (Pakistan), based on the protocol proposed by Prof. Papageorgiou [16] and was analyzed for A/S derivatives by HPLC-DAD (Agilent Technologies, Germany). Regarding pure A/S compounds (Fig. 1), commercial samples of β,β-dimethyl-acryl-shikonin (ABCR GmbH & Co., Lot AV11-XP), isovaleryl-shikonin (TCI Europe, Lot AV41-UP), acetyl-shikonin (ABCR GmbH & Co.), and deoxy-shikonin (TCI Europe, Lot AV21-VQ) were tested, which were examined for their purity by HPLC-DAD. Monomeric shikonin was purified by A. Assimopoulou from a commercial shikonin sample (Bioshikonin; Ichimaru Pharcos Co. Ltd.) by silica gel column chromatography. Its purity and identity were determined by HPLC (purity > 98%), chiral LC and LC-MS. Monomeric alkannin was isolated and purified by A. Assimopoulou from a commercial sample (Ikeda Corp.) by Sephadex LH-20 column chromatography (dichloromethane and acetone eluants). Its purity and identity were determined by HPLC (purity > 98%), chiral LC and LC-DAD-MS. Dimeric A/S was isolated and purified by A. Assimopoulou from a commercial sample (Ikeda Corp.) by a combination of chromatographic procedures (silica gel, Sephadex LH-20). Its purity and identity were determined by HPLC (purity > 98%) and LC-DAD-MS.

CA (Mn = 30,000, 39.8 wt.% acetyl content), PCL (Mn = 70,000–90,000 Da), sodium lauryl sulfate (SLS), Dulbecco’s Modified Eagle’s Medium (DMEM), fetal bovine serum (FBS), penicillin/streptomycin, trypsin-EDTA, glutaraldehyde, osmium tetroxide, filter papers (Whatman® glass microfiber filters) and 3-(4,5-Dimethylthiazol-2-yl)-2,5-diphenyltetrazolium bromide (MTT) were purchased from Sigma-Aldrich (USA). Human foreskin fibroblast Hs27 cells (ATCC® CRL-1634™) were employed in cell culture experiments and were purchased from the American Type Culture Collection (ATCC, USA). Viability/Cytotoxicity Assay Kit for Animal Live & Dead Cells was purchased from Biotium (USA). Gram-positive bacterial strains *Staphylococcus aureus* (LMG 8224) and *Staphylococcus epidermidis* (LMG 10474) were obtained from the Belgian Co-ordinated Collections of Micro-organisms (BCCM, Belgium). Nutrient agar (NA) and Muller-Hinton (MH) agar were purchased from Merck (Germany). Dimethyl-sulfoxide (DMSO) was obtained from AppliChem (Germany). Phosphate buffered saline (PBS) was purchased from Gibco® (Thermo Fisher Scientific, USA). All reagents used in the experiments were of analytical grade. N,N-Dimethyl-formamide (DMF) was purchased from Honeywell (Charlotte, North Carolina, USA), whereas acetone, dichloromethane (DCM) and ethanol were obtained from Fisher Scientific (USA).

### Preparation of polymer solutions

#### CA

CA solution was prepared at a fixed polymer concentration of 20% w/w in a binary solvent system consisting of 2:1 v/v acetone/DMF. A/S mixture, dissolved in the same solvent system (10% w/w), was afterwards added to the CA solution at various concentrations (0.05, 0.1, 1 and 5 wt.% based on polymer weight).

#### PCL

PCL solution was prepared at a fixed polymer concentration of 18% w/w in a binary solvent system consisting of 7:3 v/v DCM/DMF. A/S mixture was first dissolved in the same solvent system (10% w/w) and then it was added to the PCL solution at various concentrations (1 and 5 wt.% based on polymer weight).

### Electrospinning process

All unloaded and A/S mixture-loaded solutions (Table 1) prepared as described in Section 2.2., were allowed to stir overnight at room temperature, to obtain a homogeneous solution. For the electrospinning experiments, the apparatus used was the same as implemented by Kontogiannopoulos et al. [11] and Arampatzis et al. [15]. A more detailed description of the electrospinning setup and process can be found in the supplementary information.

**Table 1:** Composition of the electrospun fiber mats

Sample name	Polymer type	Polymer solution (% w/w)	A/S mixture (wt.%) ^**a**^
CA-n	CA	20.0	–
CA-AS005	CA	20.0	0.05
CA-AS01	CA	20.0	0.1
CA-AS1	CA	20.0	1.0
CA-AS5	CA	20.0	5.0
PCL-n	PCL	18.0	–
PCL-AS1	PCL	18.0	1.0
PCL-AS5	PCL	18.0	5.0

^a^ Based on polymer weight

### Physicochemical characterization of nanofibrous scaffolds

#### Morphology of nanofibrous scaffolds

The surface morphology of unloaded and A/S mixture-loaded nanofibers was examined by means of SEM. The samples were sputter coated with carbon to acquire electrically conductive surfaces and observed under a SEM [(mod. JSM-6390LV, JEOL, Japan) located at the Electron Microscopy Laboratory, School of Physics, Aristotle University of Thessaloniki] at an accelerating voltage of 20 kV. The average diameter size of nanofibers and their distributions were obtained through image analysis (100 diameter measurements for each sample in average), using ImageJ software (National Institutes of Health, USA). For all samples, the images were recorded at 2000× magnification and the average fiber diameter (± standard deviation, SD) and size distribution have been estimated.

#### Porosity assessment

For all scaffolds, the porosity was measured in accordance with the liquid displacement method, as described in our previous work [15]. In brief, the weight (approximately 23 mg) of three independent fiber mats from each sample was measured and immersed in a graduated cylinder containing 7 mL of ethanol (V_1_). The resulting volume was recorded as V_2_. After 10 min, the fiber mat was removed and the residual volume of ethanol was recorded as V_3_. The porosity was calculated as follows:

1 $$ Porosity\ \left(\%\right)=\left(\kern0.5em \frac{V_1-{V}_3}{V_2-{V}_3}\ \right)\times 100 $$

#### Water uptake ratio evaluation

The water uptake of the scaffolds was assessed as described in Arampatzis et al. [15]. Specifically, three independent fiber mats were cut into 1 × 1 cm pieces and weighed, recording their weight as W_0_. Then the scaffolds were immersed in 2 mL distilled water and incubated at room temperature for 24 h. After that time, their wet weight was measured again (W_1_). The water uptake ratio was calculated by the following equation:

2 $$ Water\ uptake\left(\%\right)=\left(\kern0.5em \frac{W_1-{W}_0}{W_0}\ \right)\times 100 $$

#### Thermal analysis

The thermal properties of electrospun nanofibers were examined using a differential scanning calorimeter (mod. DSC-50, Shimadzu, Japan). Measurements were carried out at a temperature range of 25–250 °C, using a heating rate of 10 °C/min, under a constant nitrogen flow of 50 mL/min, with the average sample weight being 3–5 mg.

#### Contact angle analysis

The surface wettability of the electrospun scaffolds was assessed via contact angle analysis. The measurements were carried out using a contact angle optical goniometer (mod. Cam200, KSV Instruments, Finland) located at the Laboratory of Chemical Process and Plant Design, School of Chemical Engineering, Aristotle University of Thessaloniki. Briefly, small pieces from the fiber mats were cut into 2 × 2 cm and placed on the sample stage of the instrument. Next, a drop of water was dropped from 2 cm above the surface of the scaffolds with the aid of a 500 μL threaded-plunger glass syringe (mod. 1750, Hamilton, USA). When the drop contracted the surface of the scaffolds, photographs were captured with the camera attached to the system and the contact angle was calculated by the inbuilt software of the instrument.

#### API entrapment efficiency

The actual amount of the A/S mixture (API) incorporated in the polymeric nanofibrous matrix (both inside and onto the surface of the fibers) compared to the initial amount of drug used –hereinafter referred to as entrapment efficiency – was determined by UV/Vis spectrophotometry (mod. UV-1900 spectrophotometer, Hitachi, Japan) at *λ*_*max*_ = 516 nm. In specific, drug-loaded CA and PCL nanofibers were weighed (~ 5 mg per sample) and dissolved (under vortexing) in 4 mL chloroform to dissolve the fiber structure, thus releasing the drug in the organic solvent. Subsequently, the amount of A/S mixture present in the organic phase was measured spectrophotometrically (control: chloroform; calibration curve presented in the supplementary file).

The entrapment efficiency and drug loading were calculated based on the Eqs. (3) and (4), respectively:

3 $$ Entrapment\ efficiency\left(\%\right)=\left(\ \frac{weight\ of\ drug\ entrapped\ in\ the\ fibrous\ matrix}{weight\ of\ drug\ added\ in\ the\ polymer\ solution}\ \right)\times 100 $$

4 $$ Drug\ loading\left(\%\right)=\left(\kern0.5em \frac{weight\ of\ drug\ entrapped\ in\ the\ fibrous\ matrix}{weight\ of\ fiber\  mat}\ \right)\times 100 $$

In order to assess the distribution of the drug across each fiber mat, three independent meshes were selected from random parts of the electrospun membrane and analyzed as described above. The measurement was performed in three replicates each time (*n* = 9 for each sample) and the average values (± SD) have been estimated.

#### In vitro dissolution studies

The release of the A/S mixture from electrospun nanofibers was investigated in phosphate buffer + 1% SLS at pH 5.7. Drug-loaded nanofibers (25–30 mg) were incubated in 30 mL of the release medium at 35 °C in a thermostatted shaking water bath (Bioline, Greece). At specific time intervals 3 mL aliquots were removed from the release medium and the same volume of fresh medium was replaced for maintaining sink conditions. The released A/S mixture in the dissolution medium at different timepoints was estimated using a UV/Vis spectrophotometer at 516 nm through a calibration curve (Eq. 5). Three independent fiber mats were analyzed in triplicate (*n* = 9) and the average values (± SD) have been estimated.

The cumulative percentage of A/S mixture release was calculated and plotted as a function of time, based on the following equation:

5 $$ \% Cumulative\ drug\ release=\left(\ \frac{drug\ released}{entrapped\ drug}\ \right)\times 100 $$

Calibration curve used for quantitation, is depicted in the supplementary file.

### In vitro biocompatibility assessment

#### Cell seeding

Hs27 cells were cultured in 75 cm^2^ cell culture flasks in DMEM containing 10% FBS, 100 units/mL penicillin and 100 μg/mL streptomycin and incubated at 37 °C in 5% CO_2_ atmosphere (CO_2_ Incubator, mod. MCO-19 M, Panasonic, Japan). The cell culture medium was replaced every 2–3 days. Prior to cell seeding, all fibrous scaffolds were cut into circular discs with a diameter of 8 mm, using a biopsy punch (GIMA, Italy), sterilized by UV from both sides under a laminar flow hood (Class II Biosafety Cabinet, mod. BSC-1300IIA2-X, Biobase, China) for 60 min in total, placed in 24 well plates and immersed in complete DMEM overnight. Hs27 fibroblasts (passage 5–8) were trypsinized (0.25% trypsin-EDTA), counted with a hemocytometer (BRAND GmbH, Germany) and suspended in 450 μL of complete medium, at a density of 0.5 × 10^5^ cells/disc. Next, the cells were applied on the surface of the sterile scaffolds and incubated at 37 °C with 5% CO_2_ for 40 min to facilitate cell attachment prior to addition of 1 mL/disc of complete medium.

#### Cell attachment (live/dead assay)

In order to assess the attachment of Hs27 cells on the surface of the scaffolds, confocal laser scanning microscopy was employed. For this purpose, the Viability/Cytotoxicity Assay Kit for Animal Live & Dead Cells was used, containing 2 μM Calcein-AM and 4 μM ethidium homodimer III (EthD-III). All scaffolds were PBS-rinsed and stained with Calcein-AM and EthD-III, after 3 and 7 days of culture. Calcein-AM stains living cells, whereas EthD-III dead cells (nuclei). The stained fiber mats were incubated for 30 min in the dark at room temperature and washed two times with PBS. Thereafter, a confocal upright microscope [(mod. D-Eclipse 80i C1, Nikon, Japan) located at the Laboratory of Anatomy, Histology and Embryology, Faculty of Veterinary Medicine, Aristotle University of Thessaloniki] was used for observing the samples. For the excitation of Hs27 fibroblasts, the 488 nm (for Calcein-AM) and 543 nm (for EthD-III) lasers were implemented, while the detection of the emitted light was performed at 520 nm and 617 nm, respectively. z-Stack images of the electrospun constructs were acquired with the EZ - C1 3.20 software [17]. Finally, a cell infiltration analysis was conducted on z-stack images of live cells by using the 3D surface plot plugin of ImageJ.

#### Cell morphology

The morphology of Hs27 cells on the surface of the fiber mats was observed using SEM, as described above in – the morphology of the nanofibrous scaffolds section. Scaffolds were removed from the culture medium after 3 and 7 days of incubation and washed with PBS twice. Cell fixation was accomplished with 3% v/v glutaraldehyde followed by PBS washes and post-fixation with osmium tetroxide. Once the Hs27 cells were fixed, they were dehydrated in different concentrations (30–100% v/v) of ethanol and left to dry. Subsequently, the scaffolds were sputter-coated with carbon and analyzed by SEM (as in [15]).

#### Cell viability

##### Cell-seeded scaffolds loaded with A/S mixture

In an effort to assess the metabolic activity of Hs27 cells in the presence of the A/S-loaded scaffolds, the cell viability of the seeded fibroblasts onto the nanofibers was evaluated at days 3 and 7 by the MTT colorimetric assay, as described in Arampatzis et al. [15]. Briefly, cell-seeded fiber mats were transferred to a new 24-well plate and 400 μL of the culture medium along with 40 μL of MTT at a final concentration of 0.5 mg/mL were applied in each well, followed by a 4 h-incubation at 37 °C in 5% CO_2_ atmosphere. Next, the supernatants from all wells were aspirated, 400 μL of SDS-HCl solution were added in order to dissolve the insoluble purple crystals of formazan and the plate was incubated overnight at 37 °C with 5% CO_2_. For determining cell viability, aliquots of the dissolved formazan solution (100 μL) were transferred to a 96-well plate and the absorbance was measured by a micro-plate reader (Stat Fax – 2100, Awareness Technology Inc. USA) at 570 nm. Drug-free scaffolds were considered as controls. Absorbance values were normalized with respect to the relative metabolic activity of the control (neat) samples. This means relative metabolic activity of the cells on the A/S mixture-loaded scaffolds with respect to the cells on neat scaffolds, in converting the water soluble MTT to insoluble formazan crystals. Three independent fiber mats for each sample were analyzed in triplicate (*n* = 9) and the average values (± SD) were used in calculations.

##### SAR study of A/S derivatives and their effect on Hs27

Additionally, in order to attribute the cell toxic/proliferative activity of the API-loaded scaffolds and the A/S mixture to individual compounds, the direct effect of the A/S mixture and each of the corresponding pure A/S compounds (deoxy-shikonin, monomeric alkannin and shikonin, acetyl-shikonin, isovaleryl-shikonin, β,β-dimethyl-acryl-shikonin and dimeric A/S) on the metabolic activity of Hs27 fibroblasts was investigated at different concentrations (500 nM and 1 μM), after 24 h and 3 days of cell culture. In this regard, each compound was first dissolved in DMSO and further diluted with DMEM up to the desired concentrations (< 0.1% v/v DMSO to avoid toxicity). Next, Hs27 cells cultured on 96-well plates (5000 cells/well) were treated with the above compounds at both concentrations for 24 h and 3 days. After the respective incubation times, the metabolic activity was evaluated by MTT assay as described in the previous section. Untreated cells served as controls. Results are presented as average values (± SD) of three independent experiments and relatively to the absorbance of the control samples.

#### In vitro wound healing assay

Cell migration of Hs27 fibroblasts in the presence of the electrospun scaffolds containing the A/S mixture at different drug-to-polymer ratios was assessed through the in vitro wound healing assay, as described by Augustine et al. [18]. Briefly, Hs27 cells were seeded in a 12-well plate (5 × 10^4^ cells/well) and incubated at 37 °C in 5% CO_2_ atmosphere. When the cells formed a dense monolayer, a vertical scratch was made with the aid of a sterile 100 μL pipette tip. Subsequently, the culture medium was aspirated, and cells were washed with PBS in order to remove cell debris. UV-sterilized scaffolds (8 mm in diameter) were placed in all wells and incubated for 24 h, after adding 1 mL DMEM medium per well. Cell migration was observed through an inverted microscope (mod. CKX53, Olympus, UK) and images were recorded before placing the scaffolds, as well as after 24 h. For analyzing and measuring the wound area, captured images were converted to grayscale in ImageJ (*Image ➔ Type ➔ 8-bit*) and the wound areas were selected by tracing their edges with a ‘Free Selection’ tool in the open-source software GNU Image Manipulation Program (GIMP, version 2.10.22), The GIMP Development Team (1996–2021). Next, the selected areas were imported back in ImageJ, where any cells present (in the wound areas) were highlighted by coloring their edges white (*Process ➔ Find edges*, then *Process ➔ Sharp*) and leaving the background (empty wound area) black. Finally, a manual threshold was applied (*Image ➔ Adjust ➔ Threshold*) for each sample/area and the % wound closure was calculated based on the surface coverage of the identified cells.

### Antibacterial activity

The antibacterial activity of neat and A/S mixture-loaded fibrous scaffolds was evaluated against the Gram-positive strains, *Staphylococcus epidermidis* LMG 10474 and *S. aureus* LMG 8224, implementing the Kirby-Bauer disc diffusion method [15, 19]. The bacteria were cultured onto nutrient agar plates (24 h at 37 °C) and then a dense bacterial suspension was made by transferring a small portion of staphylococci (10 μL) with an inoculating loop to 1 mL of sterile PBS. Next, 100 μL of this suspension were spread to Mueller-Hinton (MH) agar plates for conducting the test. UV-sterilized nanofibers with an average diameter of 8 mm were placed on the surface of inoculated MH plates. The plates were incubated for a period of 24 h at 37 °C. For comparison reasons and in order to elucidate which of the A/S mixture-containing derivatives – alone or in combination/synergy – exert the antibacterial activity, along with the electrospun scaffolds, filter papers were cut in 8 mm discs and loaded with various pure A/S compounds (deoxy-shikonin, monomeric alkannin and shikonin, acetyl-shikonin, isovaleryl-shikonin, β,β-dimethyl-acryl-shikonin and dimeric A/S; 30 μg/disc). After 24 h, the antibacterial activity was evaluated by measuring the inhibition zone of the bacteria in millimeters.

### Statistical analysis

Results are shown as mean value ± SD. For evaluating the statistical significance in the differences of fibers’ diameter size, porosity, water uptake capacity, entrapment efficiency, and viability of cell-seeded scaffolds and A/S compound-treated cells, one-way analysis of variance (ANOVA) along with Tukey’s post-hoc multiple comparison tests were used. Differences with *p*-value (p*) < 0.05 were considered statistically significant. All statistical analyses were performed using SPSS 25.0.

## Results

Based on the findings of our previously published works [11, 15], we were intrigued to explore the biological properties of different polymer-based electrospun scaffolds combined with bioactive A/S derivatives, in an endeavor to develop a potent dermal drug delivery system capable of promoting wound healing and skin regeneration. In this context, we selected two biodegradable and biocompatible polymers (CA and PCL) loaded with a mixture of A/S derivatives to evaluate their physicochemical and biological characteristics. According to Papageorgiou et al. [7, 9, 20], the A/S mixture –containing mainly esters– displays superior wound healing properties compared to individual, pure A/S compounds. In our study, A/S mixture has a well-defined chemical composition that is always crucial for the biological activity.

### Physicochemical characterization

#### Morphology of nanofibrous scaffolds

**Fig. 2:**
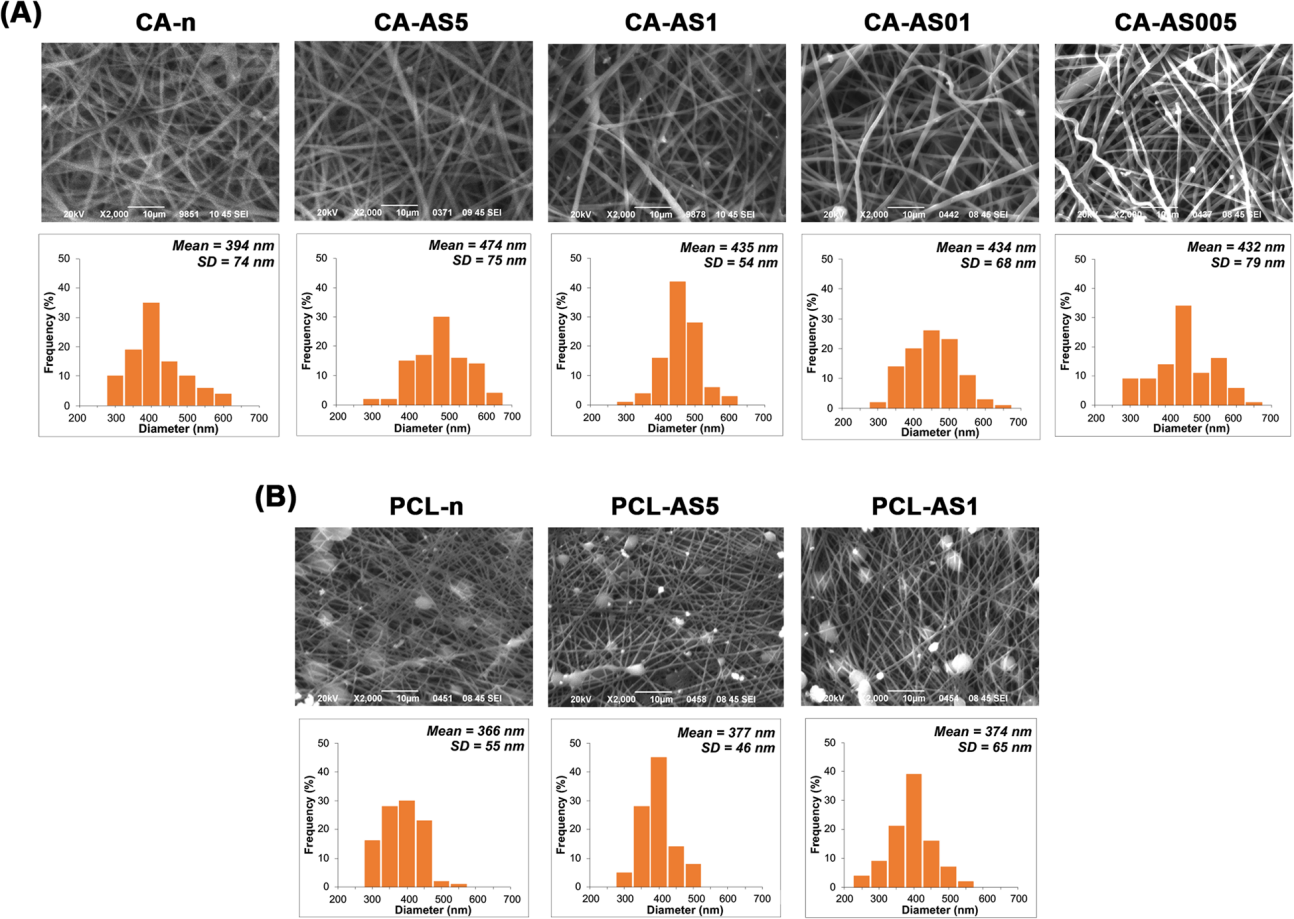
Fiber morphology of **a**) CA- and **b**) PCL- based scaffolds with varying drug-to-polymer ratios (shown in Table 1), by SEM analysis and diameter size distributions of neat and A/S mixture-loaded electrospun scaffolds. Scale bar 10 μm

**Fig. 3:**
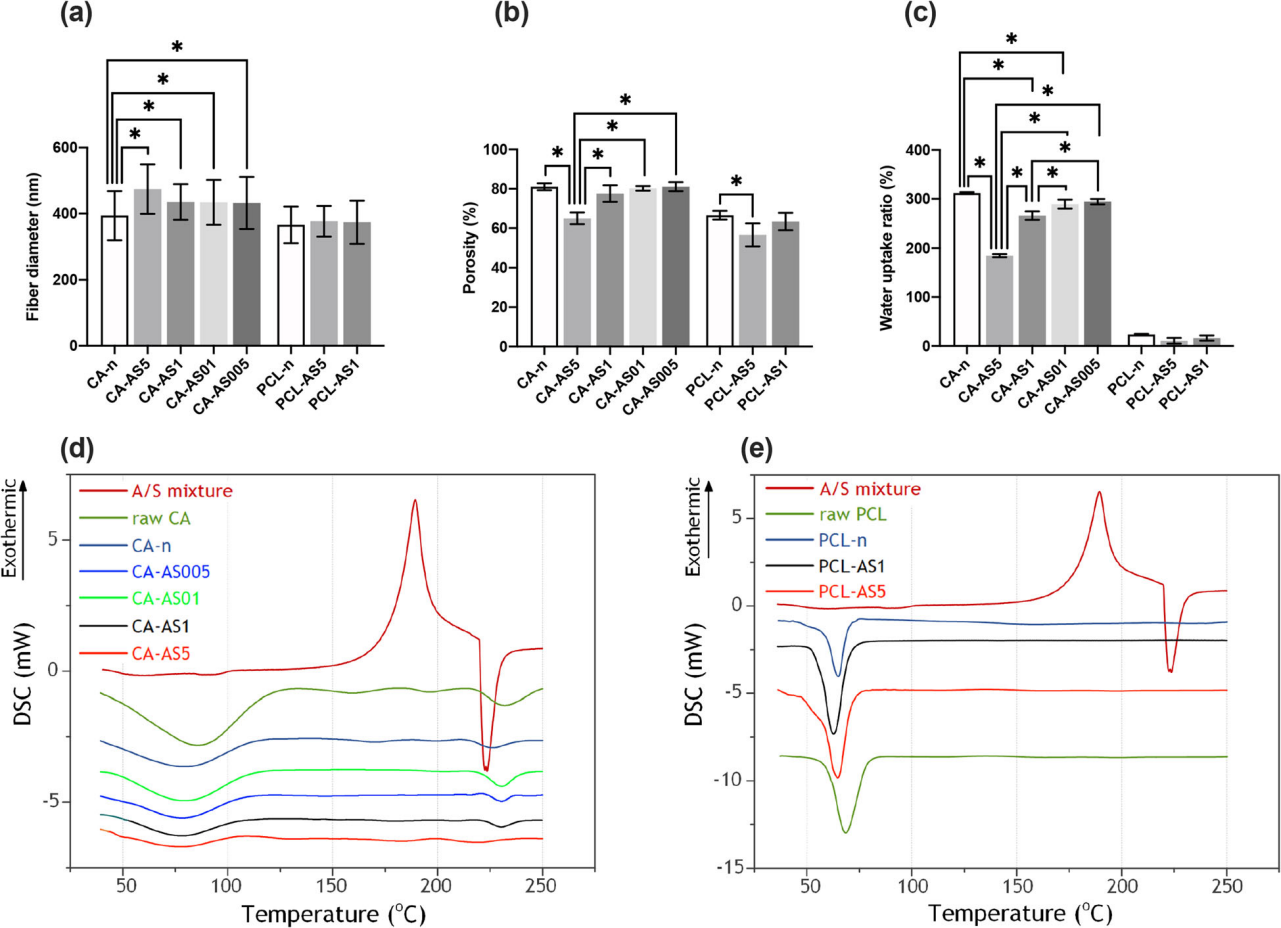
Characterization of the physicochemical properties of the CA- and PCL-based electrospun scaffolds: **a**) Fiber mean diameter, **b**) porosity (values in triplicate from three independent samples) and **c**) water uptake ratio (values in triplicate from three independent samples) analysis. Each value represents mean ± SD. * indicates statistically significant differences at *p* < 0.05. DSC thermographs for neat and A/S mixture-loaded: **d**) CA and **e**) PCL fiber mats

Figure 2 depicts the SEM micrographs of CA- and PCL-based electrospun nanofibers containing different concentrations of A/S mixture, along with their diameter size distribution histograms. All CA samples (Fig. 2a) demonstrated a defect-free and homogeneous morphology with the fiber surfaces appearing to be smooth and predominantly free of aggregates. The incorporation of the A/S mixture into the CA nanofibrous matrix led to a minor increase in fiber size, yet statistically significant (Fig. 3a). The average diameter of neat and A/S mixture-loaded nanofibers was maintained between 394 ± 74 and 474 ± 75 nm (Fig. 3a). On the contrary, electrospinning of 18% w/w PCL in DCM/DMF 7:3 v/v led to the formation of beads along the fibers (Fig. 2b). Prior to that, a series of preliminary experiments with different solvent systems and polymer concentrations had been undertaken, in order to achieve bead-free fiber mats. However, only 18% w/w PCL resulted in nanofibers with a lower number of beads. The varying wt.% of A/S mixture in the PCL-based scaffolds did not significantly change the fiber morphology with the mean fiber diameter ranging between 366 and 374 nm.

#### Porosity and water uptake ratio of the electrospun scaffolds

The % porosity of CA- and PCL-based scaffolds (Fig. 3b) ranged between 56.67 and 81.1%, with the CA-based scaffolds achieving the highest percentages. In general, the desired porosity values for tissue engineering application lies between 60 and 90% [21]. Additionally, the water uptake ratio was determined for all samples (Fig. 3c). All CA samples showed an increased water absorption capacity, in contrast to the more hydrophobic PCL nanofibers, in which the respective percentages reached only up to 23.4%. Neat CA-based scaffolds achieved a water uptake ratio higher than 300%, while the A/S mixture-loaded samples showed values between 184.64% (CA-AS5) and 294.44% (CA-AS005).

#### Thermal analysis

Figure 3d and e illustrates the DSC thermograms of the A/S mixture, the raw CA and PCL polymers and the obtained neat and drug-loaded CA and PCL electrospun scaffolds. With respect to A/S mixture, the DSC analysis showed an exothermic peak at 190 °C and an endothermic one at 225 °C. These peaks correspond to the crystallization (190 °C) and melting (225 °C) temperatures of the A/S mixture, thus exhibiting its semi-crystalline nature. In the case of raw and electrospun CA, Fig. 3d displays that the two samples share a similar broad endothermic peak between 50 °C and 100 °C. Regarding PCL, raw polymer presented an endothermic peak at 68.3 °C, corresponding to its melting point, while for the neat electrospun sample the respective peak was shifted slightly to a lower temperature (64.9 °C). Addition of the A/S mixture at 1 and 5 wt.% (nominal drug content) in the PCL nanofibers further induced the depression of melting point (62.8 °C and 64.8 °C, respectively).

#### Drug entrapment and in vitro dissolution studies

**Fig. 4:**
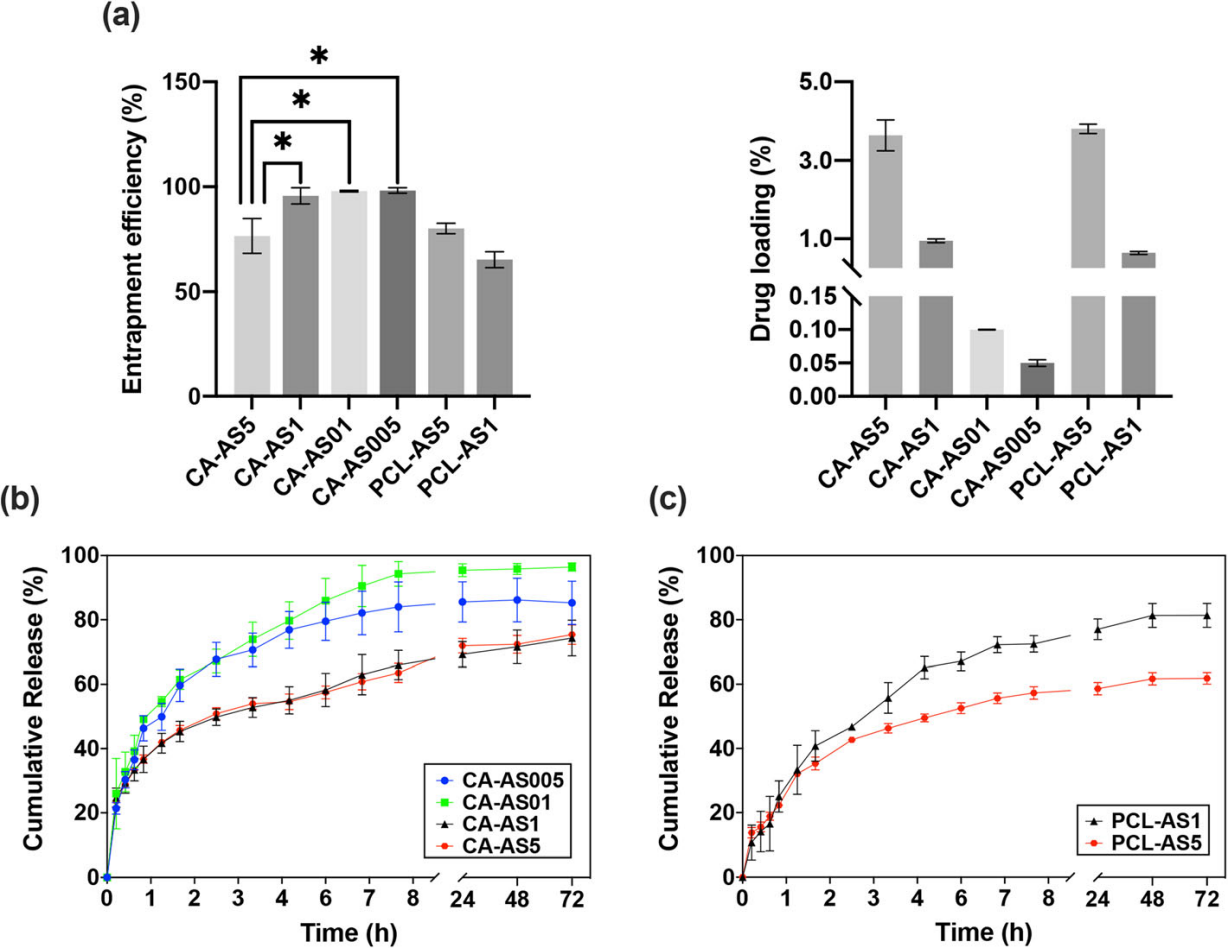
**a**) Entrapment efficiency and drug loading values estimated for A/S mixture-containing fiber mats (composition shown in Table 1). * indicates *p* < 0.05. Cumulative drug release profiles of the entrapped A/S mixture versus time from **b**) CA and **c**) PCL scaffolds. Each value represents the mean ± SD of three independent experiments (in triplicate, *n* = 9 for each sample)

Figure 4a shows the % entrapment efficiencies and the % drug loading values of the A/S mixture into the various CA and PCL nanofibers. It can be observed that for CA-based scaffolds the corresponding percentages were higher than 95%, for A/S mixture up to 1% nominal content, with CA-AS5 sample acquiring a significantly lower, yet satisfying yield of 76.48%. PCL-based scaffolds, in spite of the lower percentages, displayed an increasing trend in drug entrapment with increasing nominal drug content.

Regarding the in vitro release of A/S mixture from CA- and PCL-based scaffolds, Fig. 4b and c illustrate the cumulative release of the entrapped drug versus time. The dissolution study was carried out under sink conditions, in order to secure that the released compound would not reach its solubility limit. CA plot (Fig. 4b) demonstrates that beyond the first hour, samples containing 0.05 and 0.1 wt.% of A/S mixture release in a faster rate their content (approximately, 90% after 72 h) compared to those containing (nominally) 1 and 5 wt.% of A/S mixture, thus exhibiting a more prolonged release (approximately, 74% after 72 h). It should be noted here that if we plot the released amount of A/S mixture (in mg) versus time, the release curves are analogous to the (nominal) drug loading percentages (the higher the drug loading value, the more A/S mixture is released). PCL samples, on the other hand, demonstrated a more sustained release profile, as it can be seen from the less steep curve, in the first 2 h (Fig. 4c). Interestingly, PCL-AS1 scaffolds released a greater amount of the entrapped drug at 72 h (approximately 81%) in contrast to PCL-AS5 sample, in which the respective percentage was 62%. Last, comparing PCL- with CA-based scaffolds it can be seen that for samples with comparable drug loading values, such as PCL-AS5 and CA-AS5, the latter released the incorporated API at a faster pace (with respect to the entrapped API; Fig. 4a), reaching a higher cumulative release percentage at 72 h (approximately 75%).

#### Drug release kinetics

**Fig. 5:**
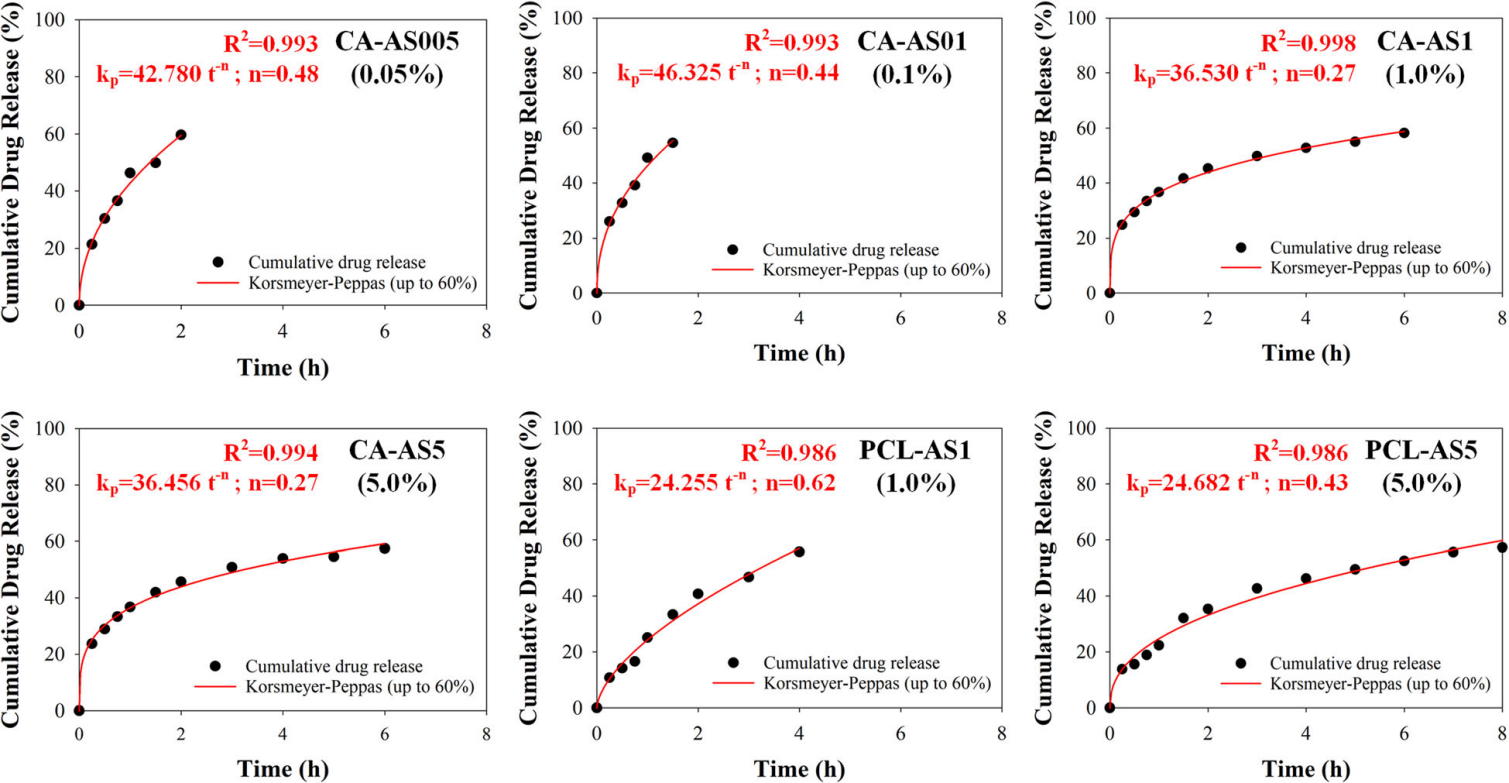
Fitting of drug release profile of all drug-loaded electrospun samples (described in Table 1) with the Korsmeyer-Peppas model, for up to 60% of totally released drug

To better understand the release kinetics of the A/S mixture from the CA- and PCL- based electrospun fiber mats, the dissolution data were fitted with the Korsmeyer-Peppas model (Fig. 5), which is a semi-empirical equation expressing drug release behavior over time and it is valid for a fractional drug release (*M*_*t*_/*M*_*∞*_) ≤ 60% [22]. The Korsmeyer-Peppas model is described by the following equation:

6 $$ {M}_t/{M}_{\infty }=k\bullet {t}^n $$

where *M*_*t*_ is the amount of drug released at time *t*, *M*_*∞*_ is the amount of drug released at infinite time, *k* is a constant related to the characteristics of the drug-polymer system, and *n* is the diffusional exponent that describes the release mechanism. The value of *n* for thin films is 0.5 for Fickian diffusion and between 0.5 and 1.0 for non-Fickian (anomalous) diffusion. In the case of cylindrical geometries of the releasing system, the exponent takes values of 0.45 for the Fickian and between 0.45 and 0.89 for the anomalous diffusion, respectively [23].

As in our previous work, where the release kinetics of A/S mixture were studied from various PHB electrospun meshes [15], the fibrous systems examined in the present study display two geometries: a cylindrical (in the sub-micron scale) and a planar (macroscopically) one. The *n* values for samples CA-AS005 and CA-AS01 were 0.48 and 0.44, respectively, whereas CA-AS1 and CA-AS5 samples exhibited even lower *n* values (0.27 for both samples). Moreover, the *k* parameter values of CA-AS1 and CA-AS5 were found to be smaller when compared with the corresponding values of CA-AS005 and CA-AS01. Regarding PCL-based nanofibers, the *n* value for PCL-AS5 was 0.43, while the respective one for PCL-AS1was higher (*n* = 0.62). Finally, the *k* values of PCL nanofibers appeared to be smaller than the respective ones of CA nanofibers.

### In vitro biocompatibility assessment

#### Cell attachment

A/S mixture-loaded nanofibers were assessed for their ability to facilitate cell attachment during the first 3 days of incubation and subsequently, cell proliferation after 7 days, on all experimental variants.

**Fig. 6:**
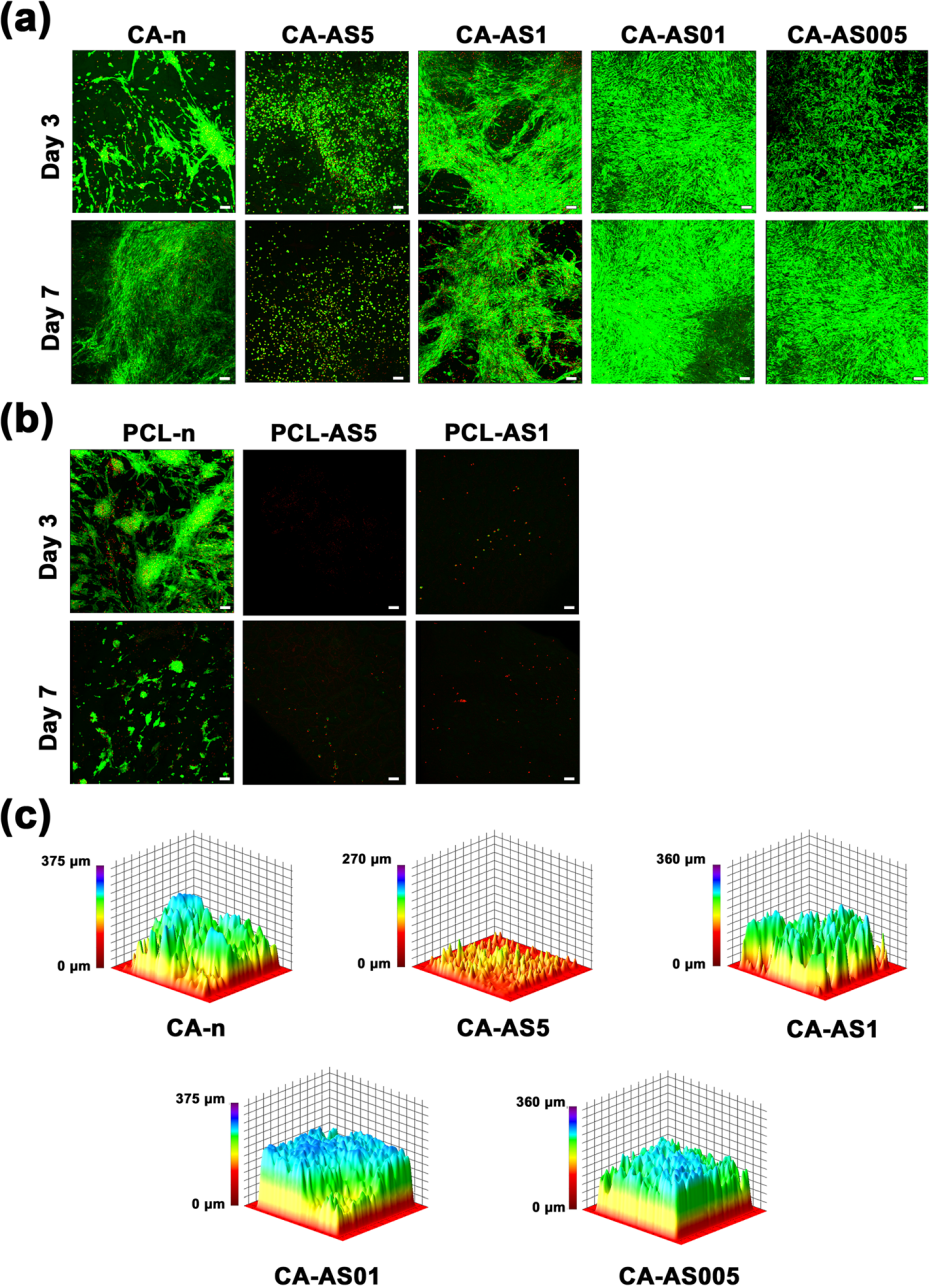
Live and dead fluorescent staining with Calcein-AM (green) and EthD-III (red) of Hs27 fibroblasts onto **a**) CA and **b**) PCL scaffolds containing different amounts of A/S mixture. Live cells are stained green, whereas dead cells (nuclei only) are stained red. **c**) Interactive 3D surface plots of Hs27 fibroblasts seeded on CA scaffolds after 7 days of culture. Confocal images were recorded at 10× magnification. Scale bar: 100 μm

The results of the confocal microscopy are presented in Fig. 6a and b. As depicted, both drug-free polymers elicited the adhesion of fibroblasts on the nanofibers. More specifically at day 3, Hs27 on neat CA scaffolds appeared to grow in clusters having elongated shapes, while after 7 days, cells proliferated forming a more tightly packed population. Accordingly, neat PCL nanofibers showed a satisfactory cell attachment at day 3 with large Hs27 colonies being formed on the entire scaffold surface. However, fewer and smaller rounded cell clusters were recorded on the scaffold surface at day 7, indicating a limited cell spreading and proliferation rate. In the case of the A/S mixture-loaded fiber mats, the morphology of the cells in CA-AS5 (days 3 & 7) indicates that the incorporation of a lipophilic drug at 5 wt.% constrained the morphology and spreading of Hs27. This observation was further validated for the more hydrophobic fiber mats, PCL-AS1 and PCL-AS5, where cell adhesion was diminished. Remarkably, when the nominal concentration of the A/S mixture in CA scaffolds was reduced to 1% (sample CA-AS1) or less (samples CA-AS005 & CA-AS01), fibroblasts – both days 3 and 7 – showed a dramatic increase regarding attachment and proliferation, especially in the case of CA-AS005 (91.52% cell surface coverage at day 7) and CA-AS01 (92.28% cell surface coverage, achieving the highest cell proliferation). Finally, analyzing the distribution of Hs27 on CA scaffolds, after 7 days, it was found that CA-AS005 and CA-AS01 fiber mats promoted not only the formation of a dense layer of cells on their surface, but also the proliferation of cells inside the nanofibrous matrix (Fig. 6c).

#### Cell morphology

Taking into account the results from cell attachment evaluation of CA and PCL nanofibers, we chose to continue with CA-based scaffolds and more specifically, with CA-n, CA-AS005, CA-AS01 and CA-AS1 samples that exhibited a satisfactory cell adhesion. We implemented such scaffolds to study their effect on the morphology of the seeded Hs27 cells on the surface of the fiber mats.

**Fig. 7:**
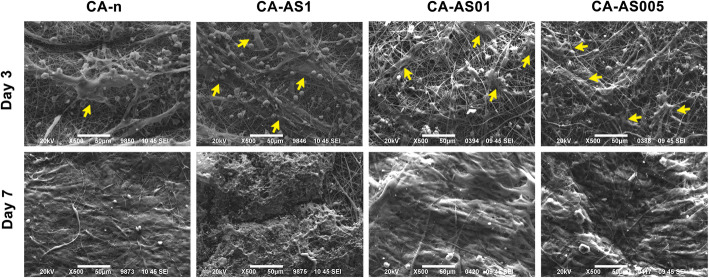
SEM micrographs of the Hs27 fibroblasts seeded on CA nanofibers (unloaded and A/S mixture-loaded) for 3 and 7 days, under 500x magnification (cells shown by yellow arrows). Scale bar: 50 μm

In support of the confocal microscopy data, SEM images (Fig. 7) evidenced the cytocompatible character of the CA-based nanofibers. After 3 days of culture, it can be observed that cells were able to adhere and proliferate on the fibrous scaffolds using their filopodia. By day 7, Hs27 cells proliferated widely on the surface of CA-n, CA-AS005 and CA-AS01 samples, with the last two exhibiting thick layers of ECM, penetrating the fiber surface and bridging the pores of the matrix.

#### Cell viability

##### Cell-seeded scaffolds loaded with A/S mixture

**Fig. 8:**
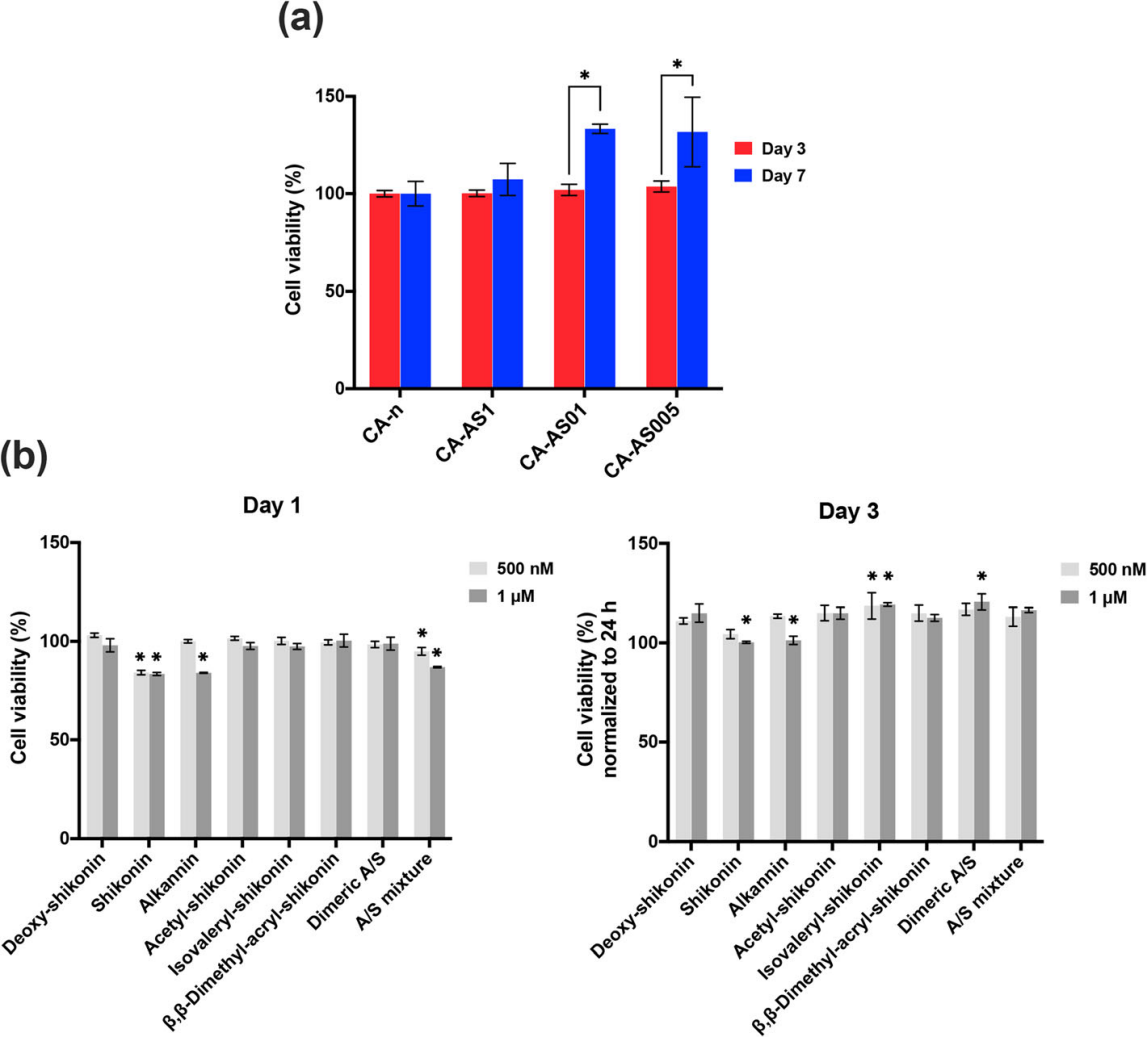
**a**) Cell viability of Hs27 cells upon seeding on A/S mixture-loaded CA scaffolds after 3 and 7 days. Cell viability values are expressed as the percentage of viability observed in neat sample (control – 100% cell viability) for the two timepoints. **b**) Cell viability of Hs27 cells upon treatment with various A/S derivatives. * indicates *p* < 0.05 compared to untreated (control) cells. Cell viability values at day 1 are expressed as the percentage of viability observed in untreated cells (100% cell viability), while cell viability percentages at day 3 are normalized based on the 24 h viability values. Data are shown as the mean of three individual experiments ± SD

The cytocompatibility of A/S mixture-loaded (nominally, 0.05, 0.1 and 1 wt.%) CA scaffolds was also investigated, in terms of cell viability by MTT assay (Fig. 8a). In particular, Hs27 cells were seeded on the surface of the fiber mats and cultured for 3 and 7 days without any signs of toxicity. Notably, all samples induced cell proliferation after 7 days, with CA-AS005 and CA-AS01 nanofibers displaying significantly increased values.

##### SAR study of A/S and their effect on Hs27

After investigating the effect of A/S mixture-containing fiber mats on Hs27 viability, we were intrigued to test the cytocompatibility of individual A/S derivatives that are present in the isolated mixture (based on HPLC-DAD analysis, the A/S mixture comprises of isovaleryl-shikonin 37.4% w/w, β,β-dimethyl-acryl-shikonin 37.24% w/w, acetyl-shikonin 20.43% w/w, deoxy-shikonin 4.01% w/w and shikonin 0.78% w/w), to attribute the activity to individual compounds.

All compounds were screened in two different time points (24 h and 3 days) in order to document both the acute and prolonged effect of A/S-exposure. Additionally, we chose relatively low concentrations of the compounds (500 nM and 1 μM), with the purpose of maintaining a non-toxic profile. Indeed, Fig. 8b illustrates that the different esters, as well as dimeric A/S, did not elicit any cytotoxicity, during the first 24 h, at any concentration. This was not the case though, for shikonin, alkannin and A/S mixture, which resulted in a significant decrease in cell number, at 1 μM (and at 500 nM for shikonin).

At day 3, all esters along with alkannin (at 500 nM), dimeric A/S and A/S mixture led to cell proliferation at both concentrations, achieving percentages higher than 110%, with isovaleryl-shikonin (at 500 nM and 1 μM) and dimeric A/S (at 1 μM) yielding the highest proliferation rates.

#### In vitro wound healing assay

**Fig. 9:**
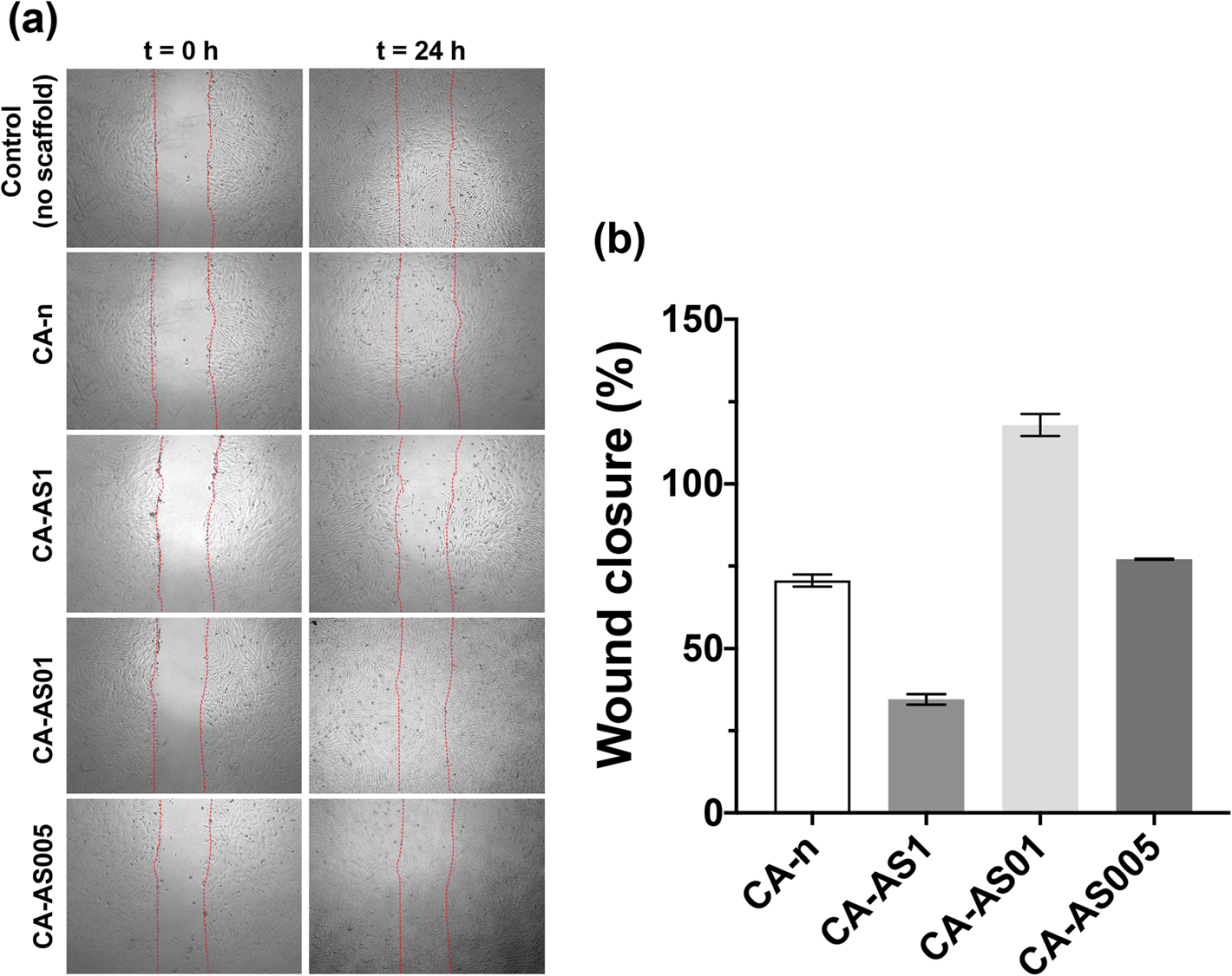
Effect of A/S mixture-containing CA scaffolds on in vitro wound healing assay using Hs27 fibroblasts. **a**) Microscopic images of the cell monolayer for the various samples, at 0 and 24 h after the wound was created. Red lines indicate the edges of the scratch performed in each condition. All images were recorded at 4× magnification. **b**) Wound closure (%) at 24 h, after treatment with the prepared scaffolds. Data are expressed as the percentage of wound closure observed in the sample where no scaffold was applied (control – 100% wound closure)

The migration of Hs27 fibroblasts in the presence of the A/S mixture-loaded CA nanofibers was evaluated after 24 h using the in vitro wound healing assay (Fig. 9).

Sample CA-AS01 containing (nominally) 0.1 wt.% of A/S mixture showed the highest wound closure of 117.9%, with CA-AS005 scaffolds demonstrating a cell migration of 77.1%, whereas the CA-n of 70.6%. CA-AS1 fiber mats exhibited an impaired scratch closure, achieving only a 34.5% contraction after 24 h.

### Antibacterial assay

#### Effect of A/S mixture-loaded nanofibers on *S. aureus* and *S. epidermidis*

The antibacterial activity of the prepared electrospun scaffolds was assessed – as in our previous work with A/S mixture-loaded electrospun PHB scaffolds [15] – against two common bacteria, *S. epidermidis* LMG 10474 and *S. aureus* LMG 8224. Both of these bacteria are highly associated with infections occurring in skin wounds and if untreated, they may potentially lead to serious complications. Table 2 highlights the observed antibacterial activity for the A/S mixture-containing CA- and PCL-based scaffolds. Higher API concentrations resulted in larger inhibition zones for both types of polymers, while for neat samples and samples loaded with less than 1% of (nominal) API, there were not any visible halos to be measured. Additionally, CA-AS1 and CA-AS5 samples demonstrated overall, a more pronounced effect towards both bacteria (compared to PCL). Τhe activity of the (high-drug content) API-loaded scaffolds can be attributed to the released A/S mixture at 24 h (as shown in Table 2) demonstrating a similar activity against both bacterial strains, achieving an average inhibition zone of 10.5 mm.

**Table 2:** Antibacterial activity against *S. epidermidis* LMG 10474 and *S. aureus* LMG 8224 at 24 h, of neat and drug-loaded electrospun CA- and PCL-based nanofibers and SAR study of pure compounds comprising the A/S mixture

			Zone of inhibition (mm)	
Sample			***S. epidermidis*** LMG 10474	***S. aureus*** LMG 8224
CA-n			–	–
CA-AS005			–	–
CA-AS01			–	–
CA-AS1			10.10 ± 0.25	9.39 ± 0.16
CA-AS5			13.21 ± 0.09	10.15 ± 1.43
PCL-n			–	–
PCL-AS1			8.71 ± 0.21	8.39 ± 0.16
PCL-AS5			10.34 ± 0.24	8.82 ± 0.74
**Compound (30 μg/disc)**	MW ^a^	Log *P* ^b^		
Deoxy-shikonin	272.3	1.08^c^	13.20 ± 0.22	12.39 ± 0.23
Shikonin	288.3	−0.27 ^c^	14.82 ± 2.12	12.34 ± 0.64
Alkannin	288.3	−0.27 ^c^	15.16 ± 0.66	12.66 ± 0.58
Acetyl-shikonin	330.3	0.66 ^c^	14.06 ± 1.25	11.87 ± 1.43
Isovaleryl-shikonin	372.4	1.77 ^c^	11.10 ± 0.94	9.13 ± 0.51
β,β-Dimethyl-acryl-shikonin	370.4	1.38 ^c^	10.59 ± 0.57	8.98 ± 0.17
Dimeric A/S	558	0.18 ^d^	9.57 ± 0.38	8.87 ± 0.30
A/S mixture	358.4 ^e^	1.35^e^	10.62 ± 0.75	10.31 ± 0.26

(−) indicates that no zone of inhibition was observed

^a^ *MW* molecular weight

^b^ Log *P*, partition coefficient in a n-octanol/water (1:1, v/v) system

^c^ Ordoudi et al. [24]

^d^ ChemDraw 20.0 Professional free trial (PerkinElmer Informatics Inc., 1985–2020) software

^e^ Calculated according to the respective values of the pure compounds and the composition of A/S mixture

#### SAR study of A/S and their effect on *S. aureus* and *S. epidermidis*

In order to attribute the antibacterial activity of A/S mixture to the individual compounds, we were urged to investigate a series of compounds, as in the previous section, that share the same basic structural moiety, but with different substitution patterns (Fig. 1).

Table 2 (as well as Fig. S2) shows that alkannin, shikonin, acetyl-shikonin, and deoxy-shikonin managed to suppress the staphylococci growth, in a diminishing order to a greater extent, than the rest of the compounds. The corresponding inhibition diameters exceeded 11.87 mm, reaching up to 15.16 mm, in the case of alkannin towards *S. epidermidis* LMG 10474. In contrast, isovaleryl-shikonin, β,β-dimethyl-acryl-shikonin, dimeric A/S and A/S mixture exhibited lower activities – for the same tested concentration – ranging between 8.87 mm and 11.1 mm.

Furthermore, in order to define whether dimerization of A/S affects the antibacterial activity, we isolated and purified a dimeric A/S derivative (structure shown in Fig. 1). Dimerization of A/S led also to a slightly decreased antibacterial activity compared to the monomeric A/S. Comparisons were performed both at μg concentrations (30 μg/disc, Table 2), as well as at μM concentrations (53 μM/disc which corresponds to 30 μg/disc for dimeric A/S and 15 μg/disc for shikonin and alkannin). For *S. epidermidis,* the inhibition zones of shikonin and alkannin were 10.77 ± 1.07 and 10.69 ± 1.01 mm respectively, while for the dimeric derivative the inhibition was measured to be 9.57 ± 0.38 mm. For *S. aureus*, shikonin and alkannin showed inhibition zones with average diameters (± SD) of 11.08 ± 0.26 and 10.74 ± 0.74 mm, respectively, whereas for the dimeric A/S, the corresponding diameter was 8.87 ± 0.30.

## Discussion

In the present study we aimed to incorporate the API in a series of electrospun fiber mats by using two well-known biomaterials (CA and PCL) and characterize their physicochemical and biological properties. Moreover, we performed SAR studies regarding the effect of different A/S compounds – comprising the above API – on Hs27 fibroblasts and two *Staphylococcus* spp., in order to attribute the activity of the bioactive wound dressings and the A/S mixture to the individual compounds.

When fabricating a scaffold to serve as a wound dressing, its morphology is one of the most critical parameters; an ideal scaffold should mimic the natural extracellular matrix (ECM) of the skin in order to facilitate cellular interaction and communication, as well as adhesion, migration and proliferation. Electrospinning is a versatile technique for producing submicron fibers with increased surface to volume ratio that resemble also the structural characteristics of the ECM [25]. In that context, the results from SEM analysis for CA (neat and drug-loaded) samples suggested that the drug was homogeneously dispersed in the fibrous meshes and support our previous findings [11], where the A/S mixture was also entrapped into CA nanofibers. PCL samples on the other hand demonstrated a beaded fiber morphology, which is usually explained by the increased surface tension, the entanglement between polymer chains and the presence of viscoelastic forces that result in the instability of the jet [26]. Han et al. [10] obtained scaffolds with similar fiber diameters by producing PCL/PTMC nanofibers containing shikonin as API.

The % porosity measurements revealed that both CA and PCL samples achieved satisfying percentages. CA-based scaffolds, though, exhibited higher values. Interestingly, in both types of polymers it was observed that by increasing drug concentration the % porosity followed a decreasing trend. A possible explanation for this might be that as the fiber diameter increases, the distance between the nanofibers becomes smaller and eventually, the fiber mat becomes more packed and less porous [27].

Another important parameter that needs to be examined for all topical dressings intended for wound healing applications, is their ability to absorb exudates and retain a moist environment for promoting cell migration and growth. To this end, the water uptake ratio experiment showed that CA samples have the capacity to absorb increased quantities of water compared to PCL samples. This can be explained by the fact that water diffuses easier into CA fiber mats – mainly due to the remaining non-esterified hydroxyl groups of the macromolecules – more water molecules get absorbed on such fibrous structures, compared to the more hydrophobic PCL-based nanofiber mats. However, the incorporation of a hydrophobic drug into the polymer matrix impedes this phenomenon leading to decreased water ratio percentages [28].

The thermal analysis of neat CA electrospun scaffold revealed that the broad endothermic peak located between 50 °C and 100 °C (which is also present in the raw CA sample) corresponds, according to Tsioptsias et al. [29], to the overall effect of various phenomena, including the esterification reaction, moisture and residual acid vaporization, while the second endothermic peak at approximately 230 °C is related to the partial decomposition of the polymer [29, 30]. Incorporation of the A/S mixture into the CA nanofibers resulted in thermograms without endothermic peaks that could be attributed to the melting peaks of the drug, indicating probably that the drug is dispersed in the polymer matrix in an amorphous state. This can also be noticed by the increasing drug concentration in CA scaffolds that leads to a much more amorphous fiber mat. The findings for PCL suggest that it turns into a more amorphous material upon its processing by means of electrospinning, while the observed depression of melting point demonstrates the integration of drug molecules inside the polymer matrix.

The results from the drug entrapment and loading measurements suggest that in the case of CA samples, A/S mixture appeared to have excellent compatibility with the matrix/solvent system, thus forcing most of the initially used drug to be integrated into the fibrous matrix. However, the quantity of the entrapped A/S mixture in the CA-AS5 sample was reduced significantly compared to the rest of the samples, showing that at higher concentrations the hydrophobic nature of A/S derivatives hinders the entrapment of greater amounts of drug or presumably, the polymeric matrix has reached its maximum drug loading capacity at the concentration of 1 wt.%. A similar percentage to the one measured for CA-AS5 was documented by our group [11], in which CA nanofibers had been implemented for incorporating A/S mixture at a percentage of 5 wt.%. For PCL samples, it can be assumed that the lower entrapment yields might be due to the physicochemical behavior of the polymer-drug solution and the corresponding jet instability that led to bead formation, as mentioned previously. It has been reported that small amounts of drug can be trapped on the interior of the syringe, tubing or spinneret and as result lower entrapment efficiencies are observed [31].

The in vitro drug dissolution study showed that CA-AS005 and CA-AS01 samples released the A/S mixture at a faster pace compared to CA-AS1 and CA-AS5. The difference observed between the low and high drug-containing scaffolds could be explained by the fact that the first ones enable to a greater extent the penetration and absorption of water molecules owing to their increased porosity. This is quite important, since the A/S mixture gets released from the polymer matrix through diffusion and thus, the more easily nanofibers get wet the higher amount of drug diffuses into the dissolution medium [32]. Nevertheless, this is also responsible for the occurrence of a burst release, during the first 2 h, together with the fact that a part of the drug molecules may be loosely bound on, or near, the surface of the fiber mats. On the contrary, PCL samples – and mainly PCL-AS5 – achieved to release their drug content in a more sustained manner. However, this resulted in releasing a smaller fraction of the entrapped drug at 72 h compared to the respective percentage of CA-AS5. Again, this supports the previous assumption that fiber mats with increased porosity become more wetted by the dissolution medium, hence the surface area of the nanofibers in contact with the medium increases and eventually, drug gets released more rapidly. These observations are in accordance with previous findings regarding the release of hydrophobic small molecules, such as chloramphenicol, from hydrophobic/hydrophilic fibers [33].

Regarding the analysis of the release kinetics of the drug-loaded electrospun scaffolds, it was observed that the CA and PCL fiber mats exhibit a cylindrical and a planar geometry. With respect to the *n* values of CA-AS005 and CA-AS01 – for the corresponding geometries – we can conclude that both of these samples release the incorporated drug following a typical Fickian diffusion. Interestingly, CA-AS1 and CA-AS5 samples demonstrated lower *n* values suggesting that the drug release is controlled also by a Fickian diffusion, but in a more sustained manner [34, 35]. This can be seen in Fig. 5 by comparing e.g. CA-AS01 (or CA-AS005) and CA-AS1 (or CA-AS5) samples, where in the case of the former, a 60% of the totally released drug is reached during the first 2 h, whereas in the case of the latter the same percentage is reached in 6 h. On top of that, the slower diffusion of drug molecules through the polymer matrix of CA-AS1 and CA-AS5 is reflected in the smaller *k* parameter values in contrast to the respective values of CA-AS005 and CA-AS01. Concerning, PCL samples, the *n* value for PCL-AS5 suggests a Fickian diffusion as the dominant release mechanism of A/S mixture. PCL-AS1, on the contrary, with a higher *n* value, indicates a non-Fickian (anomalous) diffusion, dictated by the presence of both diffusional and (polymer) relaxational phenomena [23]. Finally, the smaller calculated *k* values of PCL nanofibers (compared to the respective ones of CA nanofibers) indicate that drug release occurs at a slower pace, which is also apparent from the experimental dissolution data.

In our study, the in vitro cell attachment, morphology, viability and migration of electrospun scaffolds, were assessed. The results from the live/dead assay demonstrated the increased cytocompatibility of CA nanofibers, since Hs27 cells appeared to grow in clusters, having an elongated morphology and a good distribution, after 3 and 7 days of culture. PCL scaffolds, in contrast, did not favor the attachment and spreading of Hs27 cells, therefore exhibiting a limited cell expansion on the scaffolds’ surface. Moreover, it has been demonstrated that fibroblasts tend to interact better with moderately hydrophilic surfaces compared to hydrophobic ones [36]. The addition of the hydrophobic A/S mixture to the electrospun scaffolds at a higher concentration (e.g. 5 wt.%) led to a reduction in the spreading of the Hs27 cells on the fiber mats, which indicates that the scaffold surface is transformed into a more hydrophobic substrate (as established by contact angle measurements; Fig. S1), further impeding the proliferation and distribution of Hs27. This effect was more evident in the case of PCL samples, where the nanofibers with 1 and 5% of drug content failed to facilitate any cell attachment. These findings seem to be consistent with the results of earlier studies, in which cells infiltrated faster in CA nanofibers compared to PCL [37], therefore highlighting the advantageous characteristics of CA-based fiber mats (fiber diameter, water uptake and porosity), in contrast to those of PCL scaffolds.

SEM analysis underscored the satisfactory cell adhesion and proliferation on the surface of the CA-based scaffolds, while at the same time it was observed that Hs27 cells retained their fibroblast-like morphology and succeeded in permeating the pores of the scaffolds. In support of these findings and the ones from the confocal microscopy analysis, the cell viability of CA samples was documented, showing that CA-AS005 and CA-AS01 samples promoted cell growth and proliferation between the 3rd and 7th day of culture. The effect of shikonin on fibroblast proliferation has been reported by Lou et al. [12] and Yan et al. [38], highlighting the potential of such small molecule to promote the proliferation of skin cells, and hence wound healing.

The in vitro wound healing assay revealed that the A/S mixture-loaded electrospun CA scaffolds managed to elicit cell migration (for drug content 0.05 and 0.1 wt.%), achieving high wound closure percentages, attributed to the presence of A/S derivatives. For A/S concentration of 1 wt.% suppression of cell motility was observed, as already witnessed by confocal microscopy analysis. In previous studies, where pure A/S compounds were tested (contained in low amounts in the A/S mixture that we applied in the scaffolds), it was found that shikonin (at 1 μΜ) and deoxy-shikonin (at 1.5 and 3 μΜ) enhanced the in vitro migration of human gingival fibroblasts and keratinocytes, respectively [39, 40]. Added to that, shikonin and alkannin succeeded in accelerating (dose-dependently) the proliferation of granuloma tissue in Wistar rats with the same potency, therefore, suggesting that the absolute configuration of the hydroxyl group in the side chain of these molecules did not seem to play any important role on producing this accelerating effect. This proliferative activity may be explained by an increase in infiltrating cells (e.g. macrophages), as well as the induction of fibroblasts and collagen synthesis [41]. Nevertheless, in our case the two chiral compounds exhibited different in vitro toxic/proliferative effects towards Hs27 fibroblasts.

In order to attribute the activity of the A/S mixture-containing electrospun scaffolds, we performed a SAR study on a series of individual A/S compounds that constitute the A/S mixture and screened them for their effect on the viability of Hs27 cells. The results from the MTT assay indicate that the parent molecules, shikonin and alkannin, led to a decrease in the cell population at a concentration of 1 μM (shikonin induced cytotoxicity also at 500 nM), which could be possibly attributed to the functionality of the side chain hydroxyl group of the two enantiomers (A/S). Another group has reported that shikonin and alkannin showed non-selective toxicity towards normal cell lines (human skin fibroblasts), with the IC_50_ values being 1.2 ± 0.1 μM and 1.5 ± 0.1 μM, respectively [42]. It had also been mentioned that a commercial sample of shikonin and lithospermi radix extract induced the proliferation of human fibroblasts [39, 43]. The highest proliferation rate of Hs27 fibroblasts was achieved at day 3 by isovaleryl-shikonin (at 500 nM and 1 μM) and dimeric A/S (at 1 μM). From the above, it becomes apparent that the biological profile of each individual A/S compound contributes significantly to the overall activity of the A/S mixture. Our data suggest that CA-AS005 and CA-AS01 scaffolds had a similar effect on the viability of Hs27 fibroblasts at day 3 that was also analogous to the viability percentages when the cells were treated directly with the A/S mixture at both concentrations tested.

Aiming at enhancing the biological evaluation of A/S mixture-loaded scaffolds, we examined their effect on staphylococci. The observed antibacterial activity of the electrospun scaffolds with higher drug content is clearly correlated with the presence of A/S derivatives– above a certain concentration – confirming that these natural products have the capacity to impede *Gram* positive bacteria [9, 15]. Furthermore, the increased antibacterial effect of CA-AS1 and CA-AS5 samples against both bacteria (compared to PCL) suggests that this type of polymeric fiber mats released a greater amount of their drug-content at 24 h when the antibacterial activity was assessed. Finally, the results revealed that *S. epidermidis* LMG 10474 proved to be more susceptible to A/S mixture-loaded nanofibers than *S. aureus* LMG 8224 in a concentration-dependent manner, exhibiting larger inhibition zones.

Interestingly, we observe that the antibacterial activity of the A/S mixture-containing CA scaffolds becomes evident at 1 wt.% of drug content or higher (CA-AS1 and CA-AS5). On the other hand, we have reported that CA samples with lower drug content (CA-AS005 and CA-AS01) resulted in an enhanced cell migration during the wound healing assay. These findings could be rather insightful for designing the next steps (potentially, an in vivo study). Meaning that dressings with a higher drug amount could release a large portion of their content during the early phase of the wound healing process, in order to deliver an antibacterial and anti-inflammatory effect, protect the wound from any complications and help proceeding to the proliferative phase. As the healing process progresses, lower quantities of the gradually released drug would result in an enhanced re-epithelization and tissue formation, until the complete treatment of the wound.

Furthermore, we tried to attribute the antibacterial activity of the API-loaded scaffolds to the individual A/S derivatives. From the findings of the SAR study it becomes apparent that different substitution patterns on the parent A/S moiety (presence or not of the hydroxyl group, type of ester substitution, dimerization) with varying physico-chemical properties and steric effects, lead to the modification of their antibacterial activity. It has already been proposed that the antimicrobial properties of A/S may be assigned to the generation of a semiquinone radical anion in the naphthazarin moiety [9], while dimerization of these hydroxynaphthoquinones has been found to affect their biological activity [44]. Correspondingly, the observed small decrease in the antibacterial effect of dimeric A/S may be associated with the coupling mechanism of the naphthoquinones and the related steric constraints [44]. Previous in vitro studies have reported that shikonin and acetyl-shikonin inhibited the growth of *S. aureus;* whereas β,β-dimethyl-acryl-shikonin was effective against *S. aureus* and *S. epidermidis* strains [45, 46].

Our results suggest that the presence of the hydroxyl group in the side chain of A/S enhances the antibacterial activity against staphylococci and that the activity is also retained, even when the hydroxyl group is substituted by a hydrogen or an acyl group. Moreover, when the complexity of the fatty acid ester group increases, the antimicrobial activity seems to decrease slightly, which may be explained by the increase in lipophilicity of the ester derivatives isovaleryl-shikonin and β,β-dimethyl-acryl-shikonin (Log *P* values of 1.77 and 1.38, respectively), as well as probably steric effects. The order of antibacterial activity follows almost the same pattern as the antioxidant one [24]. According to [47], lipophilicity plays a significant role in the antibacterial activity of a compound, since a highly hydrophilic molecule would never cross the cytoplasmic membrane and a highly hydrophobic molecule would bind too strongly to the phospholipid bilayer.

Based on the above findings, the antibacterial activity of the A/S mixture can be interpreted by taking into account its composition (main constituents: isovaleryl-shikonin, β,β-dimethyl-acryl-shikonin, acetyl-shikonin, deoxy-shikonin and shikonin) and the corresponding values for each compound constituting the A/S mixture. In this regard, the analysis presented herein is quite critical, considering the great chemodiversity observed not only in different *Alkanna* species, but also within the same species [48, 49], since the biosynthesis of these molecules is highly diverse, depending on the plant material, the climate/growth conditions and other parameters that are being evaluated by the MICROMETABOLITE project (http://micrometabolite.eu/). In any case, the assessment of the composition of each API used for medicines, medical devices and/or dermocosmetics, as well as for each sample evaluated for biological activity, is really crucial for the activity itself and the attribution of that to the individual compounds.

## Conclusions

The present study contributes to the continuously evolving field of skin tissue engineering through the development of biodegradable and biocompatible, non-woven wound dressings loaded with the bioactive secondary metabolites, A/S and their derivatives. These molecules have been long established as wound healing agents with strong antimicrobial and anti-inflammatory action, and so far, they have been used as APIs – discovered by our group – in a variety of well-approved medicines – targeting wound healing. The aim of this work was to exploit the already acquired knowledge from our previous studies [11, 15], harness the biological potential of the natural products A/S by incorporating them in different biomaterials and take a step forward to a potential wound care product by assessing their in vitro cytocompatibility and antibacterial activity. In that respect, we fabricated CA and PCL sub-micron fibers (< 500 nm) containing a mixture of A/S at different ratios. CA nanofibers exhibited a more favored fiber morphology and physicochemical profile with higher values of porosity and water uptake capacity compared to PCL; quite important characteristics for a wound dressing, in order to facilitate the adsorption and proliferation of dermal fibroblasts and remove wound exudates. All scaffolds showed satisfactory drug dispersion within the polymer matrix, entrapment efficiency values and appropriate drug release profiles. The in vitro biocompatibility study indicated that CA nanofibers containing A/S mixture < 1 wt.% increased adhesion, proliferation, infiltration and migration of Hs27 fibroblasts and demonstrated overall, a good cytocompatibility profile, compared to neat CA and PCL (neat/drug-loaded) scaffolds. The SAR of different A/S compounds and their impact on cell viability revealed that the ester derivatives (main constituents of the A/S mixture), as well as dimeric A/S, promoted cell proliferation after 3 days, even at low concentrations. Τhe parent compounds (monomeric shikonin at both concentrations and alkannin at 500 nM) resulted in toxicity towards the corresponding cell line, due to the side chain’s hydroxyl group. Finally, CA and PCL scaffolds containing 1 and 5 wt.% A/S mixture exhibited potent antimicrobial activity against *S. epidermidis* and *S. aureus* attributed to the A/S mixture. Approximating a SAR antibacterial study of the constituents of the A/S mixture, we found that the presence of the side chain’s hydroxyl group of A/S enhances the antibacterial activity against both staphylococci tested, while the activity is retained even with a hydroxyl group substitution by a hydrogen or an acyl group. Increased lipophilicity of A/S derivatives together probably with steric hindrance led to a slightly diminished activity against the bacteria. Altogether, for the first time we documented the remarkable biocompatibility of A/S in combination with CA nanofibers, underscoring their use as an approach for enhanced wound healing. The results obtained from this work will allow us to advance our research to in vivo studies, while at the same time envisage that our cost-effective fiber mats loaded with the regenerative agents alkannins and shikonins, can be the next generation of wound dressings to access the wound care market.

## Supplementary Information

**Additional file 1 **: **Fig. S1**. Contact angle measurement for neat and A/S mixture-loaded electrospun CA and PCL scaffolds. **Fig. S2**. Kirby-Bauer disc diffusion method for evaluating the antibacterial activity of CA- and PCL-based electrospun scaffolds (neat and A/S mixture-loaded), as well as various pure A/S compounds, against *S. aureus* LMG 8224 (A) and *S. epidermidis* LMG 10474 (B).

## Data Availability

All data generated or analyzed in this study are included in this published article.

## Abbreviations

ANOVA: Analysis of variance
API: Active pharmaceutical ingredient
A/S: Alkannin/ Shikonin
*A. tinctoria*: *Alkanna tinctoria*
AUTh: Aristotle University of Thessaloniki
CA: Cellulose acetate
Calcein-AM: Calcein acetoxymethyl ester
DCM: Dichloromethane
DMEM: Dulbecco’s modified eagle medium
DMF: N,N- Dimethyl-formamide
DMSO: Dimethyl-sulfoxide
DSC: Differential scanning calorimetry
ECM: Extracellular matrix
*E. coli*: *Escherichia coli*
EtD-III: Ethidium homodimer III
GIMP: GNU image manipulation program
HPLC: High-performance liquid chromatography
HPLC-DAD: High-performance liquid chromatography with diode-array detection
Hs27: Human foreskin fibroblast cells
LC: Liquid chromatography
LC-DAD-MS: Liquid chromatography with diode array detection-mass spectrometry
LC-MS: Liquid chromatography-mass spectrometry
*L. erythrorhizon*: *Lithospermum erythrorhizon*
Log *P*: Partition coefficient in a n-octanol/water (1:1, v/v) system
MH: Muller-Hinton
MTT: 3-(4,5-dimethylthiazol-2-yl)-2,5-diphenyltetrazolium bromide
MW: Molecular weight
NA: Nutrient agar
PBS: Phosphate buffered saline
PCL: Poly (ε-caprolactone)
PHB: Poly[(R)-3-hydroxybutyric acid]
PLGA: Poly(lactic-co-glycolic acid)
PLLA: Poly(lactic-L-acid)
PTMC: Poly(trimethylene carbonate)
PVA: Polyvinyl alcohol
SAR: Structure-activity relationship
*S. aureus*: *Staphylococcus aureus*
SD: Standard deviation
SDS-HCl: Sodium dodecyl sulfate-hydrochloric acid
SEM: Scanning electron microscopy
*S. epidermidis*: *Staphylococcus epidermidis*
SLS: Sodium lauryl sulfate
Trypsin-EDTA: Trypsin ethylene-diamine tetra-acetic acid
UV/Vis: Ultraviolet/visible
*λ*_*max*_: Maximum absorption wavelength
