# Supplementary material for: Electrospun wound dressings containing bioactive natural products: physico-chemical characterization and biological assessment
Source: Biomater Res. 2021 Jul 16;25:23. doi: 10.1186/s40824-021-00223-9 (PMC8284004; doi:10.1186/s40824-021-00223-9)
Supplement: Supplementary file 1 — Additional file 1 : Fig. S1. Contact angle measurement for neat and A/S mixture-loaded electrospun CA and PCL scaffolds. Fig. S2. Kirby-Bauer disc diffusion method for evaluating the antibacterial activity of CA- and PCL-based electrospun scaffolds (neat and A/S mixture-loaded), as well as various pure A/S compounds, against S. aureus LMG 8224 (A) and S. epidermidis LMG 10474 (B). [file 40824_2021_223_MOESM1_ESM.docx]

**Electrospun wound dressings containing bioactive natural products:**

**Physico-chemical characterization and biological assessment**

Athanasios S. Arampatzis^1,2^, Konstantinos N. Kontogiannopoulos^1,2^, Konstantinos Theodoridis^3,4^, Eleni Aggelidou^3,4^, Angélique Rat^5^, Anne Willems^5^, Ioannis Tsivintzelis^6^, Vassilios P. Papageorgiou^1,2^, Aristeidis Kritis^3,4^, Andreana N. Assimopoulou^1,2, *^

^1^ Laboratory of Organic Chemistry, School of Chemical Engineering, Aristotle University of Thessaloniki (AUTh), Thessaloniki 54124, Greece

^2^ Natural Products Research Center of Excellence (NatPro-AUTh), Center of Interdisciplinary Research and Innovation of Aristotle University of Thessaloniki (CIRI-AUTh), Thessaloniki 57001, Greece

^3^ Department of Physiology and Pharmacology, School of Medicine, Faculty of Health Sciences, Aristotle University of Thessaloniki (AUTh), Thessaloniki 54124, Greece

^4^ cGMP Regenerative Medicine Facility, Department of Physiology and Pharmacology, School of Medicine, Faculty of Health Sciences, Aristotle University of Thessaloniki (AUTh), Thessaloniki 54124, Greece.

^5^ Laboratory of Microbiology, Faculty of Sciences, Ghent University, Ghent 9000, Belgium

^6^ Physical Chemistry Laboratory, School of Chemical Engineering, Aristotle University of Thessaloniki (AUTh), Thessaloniki 54124, Greece

*Correspondence address. Organic Chemistry Laboratory, School of Chemical Engineering, Aristotle University of Thessaloniki, Thessaloniki 54124, Greece. Tel. +30 2310 994242; email: [adreana@cheng.auth.gr](mailto:adreana@cheng.auth.gr)

**Supplementary experimental procedures**

**Electrospinning process**

Each polymer solution was poured into a glass syringe (10 mL) fitted with a blunt needle of inner diameter 1 mm (18G). The flow rate for all solutions was kept at a fixed speed of 0.7 mL/h with the use of a syringe pump (mod. 2274, Harvard Apparatus). The needle was connected to the positive electrode of a high-voltage supply (mod. RHR30P30, Spellman High Voltage DC SUPPLY), whereas the ground electrode was connected to a rotating drum-collector covered in aluminum foil. The tip-to-collector distance was 12 cm and the applied voltage was 16.5 kV and 18.5 kV for CA and PCL, respectively. Once all electrospun membranes had been fabricated, they were dried under vacuum (320 Pa, Shanghai Laboratory Instrument Work Co. Ltd., China) for at least 24 h at 40 ^o^C to remove any residual solvent and stored in air-tight containers at room temperature.

**API entrapment efficiency**

For estimating drug content, a calibration curve of various concentrations (n=9) of the A/S mixture in chloroform *versus* absorbance values was constructed:

$\text{Drug concentration (mg/mL) = 0.0411 }\text{×}\text{ absorbance - 0.0002; (}\text{R}^{\text{2}}\text{=0.996)}$ (1)

***In vitro* dissolution studies**

For estimating the amount of released A/S mixture in the dissolution medium, a calibration curve of various concentrations (n=9) *versus* absorbance values was constructed:

$\text{Drug concentration (mg/mL) = 0.0392}\text{×}\text{ absorbance + 0.0001; (}\text{R}^{\text{2}}\text{=1)}$ (2)

**Supplementary results**


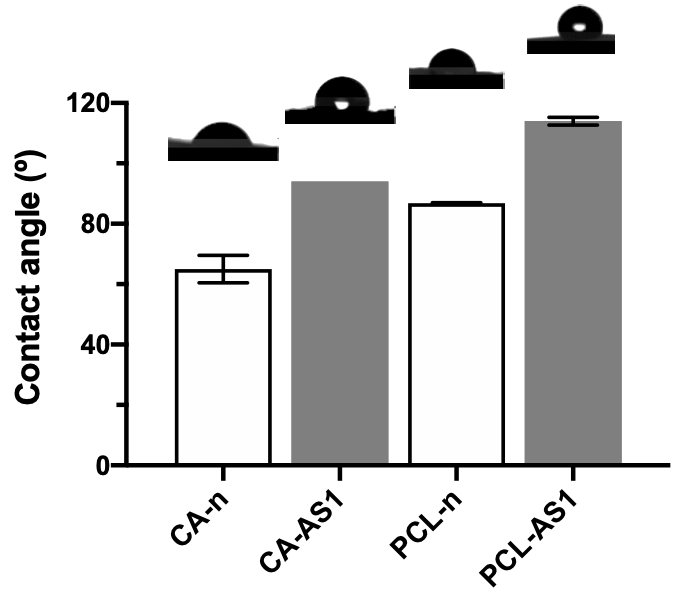


Fig. S1. Contact angle measurement for neat and A/S mixture-loaded electrospun CA and PCL scaffolds.


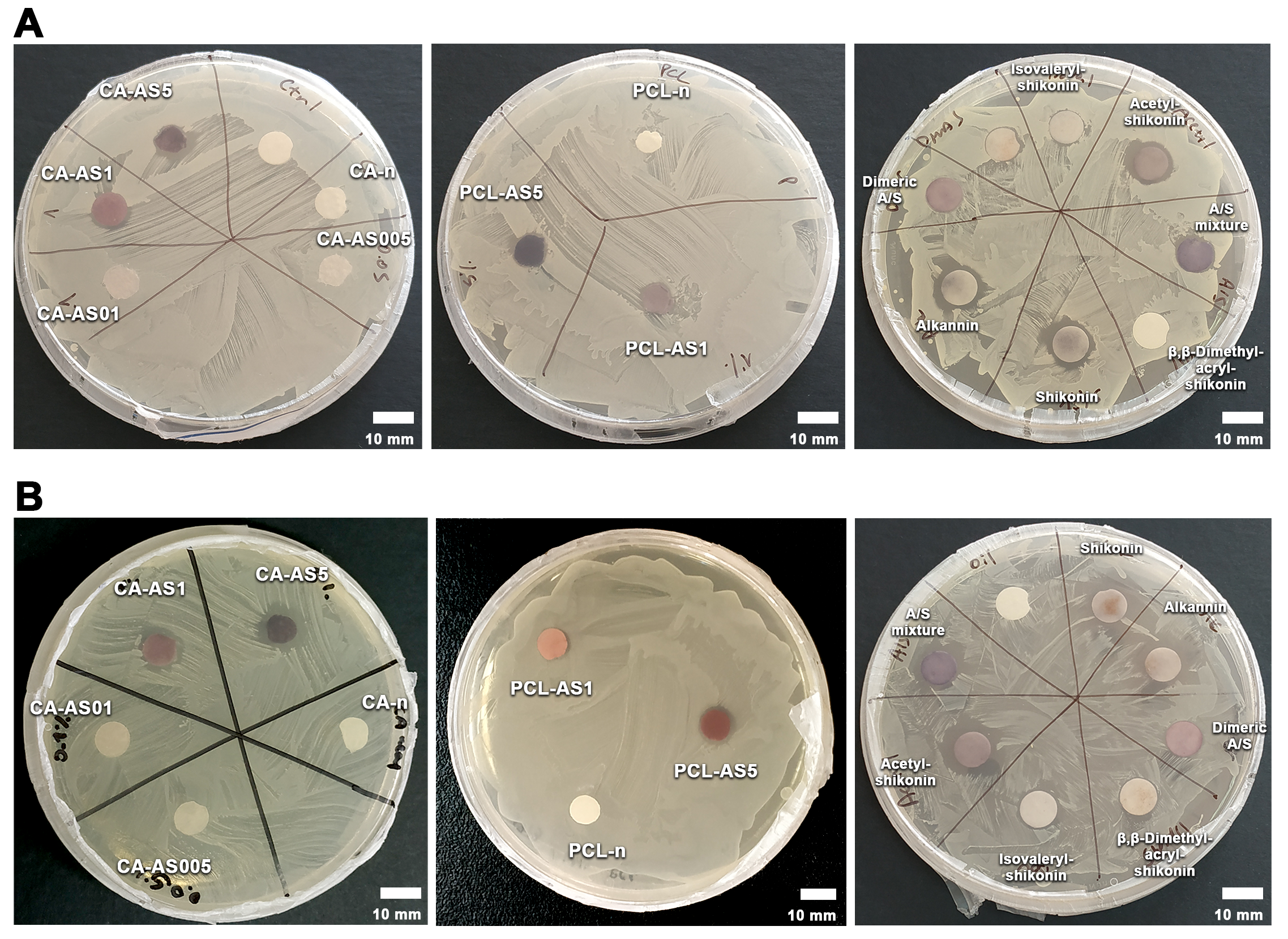


Fig. S2. Kirby-Bauer disc diffusion method for evaluating the antibacterial activity of CA- and PCL-based electrospun scaffolds (neat and A/S mixture-loaded), as well as various pure A/S compounds, against *S. aureus* LMG 8224 (A) and *S. epidermidis* LMG 10474 (B).
